# Fine-tuning the metabolic rewiring and adaptation of translational machinery during an epithelial-mesenchymal transition in breast cancer cells

**DOI:** 10.1186/s40170-020-00216-7

**Published:** 2020-07-19

**Authors:** Tamara Fernández-Calero, Marcos Davyt, Karen Perelmuter, Cora Chalar, Giovana Bampi, Helena Persson, Juan Pablo Tosar, Völundur Hafstað, Hugo Naya, Carlos Rovira, Mariela Bollati-Fogolín, Ricardo Ehrlich, Gilles Flouriot, Zoya Ignatova, Mónica Marín

**Affiliations:** 1 Biochemistry-Molecular Biology Section, Faculty of Sciences, Universidad de la República, Iguá 4225, CP 11400 Montevideo, Uruguay; 2 Bioinformatics Unit, Institut Pasteur Montevideo, Mataojo, 2020 Montevideo, Uruguay; 3 Departamento de Ciencias Exactas y Naturales, Universidad Católica del Uruguay, Av. 8 de Octubre, 2738 Montevideo, Uruguay; 4 Cell Biology Unit, Institut Pasteur Montevideo, Mataojo, 2020 Montevideo, Uruguay; 5 Institute for Biochemistry and Molecular Biology, Department of Chemistry, University of Hamburg, Hamburg, Germany; 6 Department of Clinical Sciences Lund, Oncology and Pathology, Lund University Cancer Center, Lund University, SE-223 63 Lund, Sweden; 7 Functional Genomics Unit, Institut Pasteur de Montevideo, Mataojo, 2020 Montevideo, Uruguay; 8 Analytical Biochemistry Unit, Nuclear Research Center, Faculty of Science, Universidad de la República, Montevideo, Uruguay; 9 Institut Pasteur de Montevideo, Montevideo, Uruguay; 10 Université de Rennes 1-IRSET, Campus Santé de Villejean, 35000 Rennes, France

**Keywords:** Breast cancer, EMT, Luminal to basal transition, MKL1/actin signaling pathway, Metabolism adaptation, Translation machinery, Ribosome profiling, Breast cancer stem cells

## Abstract

**Abstract:**

**Background:**

During breast cancer progression, the epithelial to mesenchymal transition has been associated with metastasis and endocrine therapy resistance; however, the underlying mechanisms remain elusive. To gain insight into this process, we studied the transition undergone by MCF7-derived cells, which is driven by the constitutive nuclear expression of a MKL1 variant devoid of the actin-binding domain (MKL1 ΔN200). We characterized the adaptive changes that occur during the MKL1-induced cellular model and focused on regulation of translation machinery and metabolic adaptation.

**Methods:**

We performed a genome-wide analysis at the transcriptional and translational level using ribosome profiling complemented with RNA-Seq and analyzed the expression of components of the translation machinery and enzymes involved in energy metabolism. NGS data were correlated with metabolomic measurements and quantification of specific mRNAs extracted from polysomes and western blots.

**Results:**

Our results reveal the expression profiles of a luminal to basal-like state in accordance with an epithelial to mesenchymal transition. During the transition, the synthesis of ribosomal proteins and that of many translational factors was upregulated. This overexpression of the translational machinery appears to be regulated at the translational level. Our results indicate an increase of ribosome biogenesis and translation activity. We detected an extensive metabolic rewiring occurring in an already “Warburg-like” context, in which enzyme isoform switches and metabolic shunts indicate a crucial role of HIF-1α along with other master regulatory factors. Furthermore, we detected a decrease in the expression of enzymes involved in ribonucleotide synthesis from the pentose phosphate pathway. During this transition, cells increase in size, downregulate genes associated with proliferation, and strongly upregulate expression of cytoskeletal and extracellular matrix genes.

**Conclusions:**

Our study reveals multiple regulatory events associated with metabolic and translational machinery adaptation during an epithelial mesenchymal-like transition process. During this major cellular transition, cells achieve a new homeostatic state ensuring their survival. This work shows that ribosome profiling complemented with RNA-Seq is a powerful approach to unveil in-depth global adaptive cellular responses and the interconnection among regulatory circuits, which will be helpful for identification of new therapeutic targets.

## Introduction

Breast cancer is the most common cancer in women worldwide and the second most common cause of cancer mortality. More than 90% of breast cancer-related mortalities are caused by its metastases at distant sites [1]. During cancer progression, cells acquire new abilities and switch from a well-differentiated epithelial phenotype to a metastatic one [2].

The epithelial to mesenchymal transition (EMT) is an essential biological process during normal development, which is also observed in cancer and other pathologies [3]. During this transition, epithelial cells lose differentiated characteristics such as cell adhesion and polarity and acquire immature features, including high cellular plasticity, motility, invasiveness, and resistance to apoptosis [4]. Cells undergoing an EMT downregulate epithelial markers, such as E-cadherin, claudins, occludins, and cytokeratins, and upregulate mesenchymal markers, such as S100A4 (also called FSP1, for Fibroblast-Specific Protein 1), vimentin, and N-cadherin [5]. In tumorigenic processes, EMTs have been found to contribute to invasion, metastatic dissemination, and the acquisition of therapeutic resistance [3]. The EMT may be incomplete and the cell population heterogeneous; hence, only part of the EMT markers may be expressed in small sets of cancer cells [5]. Among breast cancer subtypes, luminal tumors appear to have cells solely on the epithelial edge of the EMT spectrum while basal-like tumors are more heterogeneous with cells spanning the spectrum from potential progenitors to mesenchymal-oriented variants [6].

Different pathways can drive an EMT. The primary mediators of the EMT include signaling through TGF-β, Notch, and Wnt, but the transition is also influenced by the tumor microenvironment, such as hypoxia and differential expression of microRNAs, e.g., miR-200 [7, 8]. The differential expression of the transcriptional factors SNAI (Snail), Zeb, and Twist, which are common to several pathways, or silencing of the ER, may also lead to the EMT [7]. In breast cancer cells, the MKL1/actin signaling pathway drives an EM transition. The MKL1 pathway is active in breast cancer cells with a basal-like phenotype and silenced in luminal ER-positive cell lines. This member of the myocardin-related transcription factor family is a coactivator of serum response factor (SRF). MKL1 (also known as MRTFA) is a master regulator of actin dynamics and cellular motility functions. In the cytoplasm, MKL1 binds free actin monomers. Upon actin polymerization, MKL1 dissociates and translocates into the nucleus where it binds SRF and promotes the induction of SRF target genes that are involved in motile cell functions. Indeed, MKL1 and SRF are required for tumor cell invasion and metastasis. Kerdivel et al. [9] showed that the nuclear localization of ER and MKL1 in breast cancer cells is mutually exclusive. Activation of the MKL1/actin pathway in estrogen-sensitive breast cancer cells leads to hormonal resistance associated with a severe decrease in the expression of ER, PR, and HER2 [9].

Using a tetracycline-inducible expression vector system, Flouriot et al. [10] developed MCF7 subclones expressing truncated forms of MKL1. In MKL1 ΔN200 cells, the expression of the MKL1 variant with N-terminal deletion—devoid of the actin-binding sites (RPEL motifs)—leads to a constitutive activity and permanent translocation into the nucleus of this cofactor [9, 10]. The control cell line corresponds to MCF7 cells stably transfected with the empty vector. In addition, so-called MKL1 ΔC301 cells expressing MKL1 that are devoid of 301 residues from the C-terminal transactivation domain is also taken as a control cell line. Therefore, the MKL1 ΔN200 cell line appears to be a promising cellular model to address the adaptive changes that occur during breast cancer progression in an EM-like transition.

To gain detailed insights into breast cancer progression, here, we characterized the adaptive changes that occur during the MKL1-induced EM-like transition. We employed the MKL1-inducible cellular model (MCF7 control, MKL1 ΔN200, MKL1 ΔC301) and focused on regulation of translation machinery and metabolic adaptation. Leveraging the depth of NGS-based approaches, we performed a genome-wide analysis at the transcriptional and translational level using ribosome profiling complemented with RNA-Seq and analyzed the expression of components of the translation machinery and enzymes involved in energy metabolism. These data were correlated with metabolomic measurements, western blot, and quantification of specific mRNAs extracted from polysomes. This approach revealed that MKL1 ΔN200 cells exhibit features corresponding to a transition state from a luminal to a basal-like phenotype, with stem cell-like traits.

## Materials and methods

### Cell culture, glucose consumption, lactate production, and differences between cell sizes

Stably transfected MCF7 T-Rex subclones (T-Rex system, Invitrogen), MCF7-control, MCF7-MKL1ΔN200, and MCF7-MKL1ΔC301 were previously described by [9–11]. The cells were routinely maintained in DMEM Gibco™ GlutaMAX™, containing 4.5 g/L glucose and phenol red, supplemented with 10% fetal calf serum (FCS) (Gibco™), zeocin (100 μg/mL), and blasticidin (5 μg/mL), at 37 °C in 5% CO_2_. Before any experiments, MCF7 cells were maintained in phenol red-free DMEM (Thermo) supplemented with 2.5% charcoal-stripped FCS (Capricorn), 1% pyruvate (Invitrogen), and 1% l-glutamine (Invitrogen) for 48 h. To induce expression of MKL1of MKL1 protein variants (MKL1 ΔN200 and MKL1 ΔC301), MCF7 subclone cultures were treated with 1 μg/mL tetracycline for 48 h. To evaluate glucose consumption and lactate production in MCF7 subclones, cell growth curves were performed and maintained for 120 h. Cell viability was determined every 24 h by trypan blue dye exclusion method by counting viable cells using Neubauer chambers. Glucose and lactate concentrations were measured every 24 h in the supernatant using the glucose/lactate analyzer BioProfile Basic 2 (Nova Biomedical, USA). To evaluate differences in cell size during growth, the cells were analyzed using an Accuri C6 (BD, USA) flow cytometer equipped with 488 nm and 633 nm lasers. The BD Accuri C6 software was used for data acquisition and analysis. For each sample, 5000 counts gated on a Forward Scatter (FSC) versus Side Scatter (SSC) dot plot, excluding doublets, were recorded. The median of the FSC channel (FSC-A) was compared between cell lines.

### Polysome profiling

Approximately 2.2 × 10^6^ cells were seeded in 10-cm diameter plates and cultured for 24 h in DMEM and 10% FBS. Media were then changed to DMEM/F-12 (Gibco 11039-021), 2.5% charcoal-stripped FBS, and tetracycline at a final concentration of 1 μg/mL. Cells were incubated for 48 h before being subjected to polysome fractionation. Polysomal fractionation was done as described [11] with some modifications. For each sample, 190 μl was layered directly onto the sucrose gradient. These were centrifuged for 2.5 h at 37,000 rpm at 4 °C. RNA was extracted using Direct-Zol RNA Miniprep (Zymo Research). Before RNA extraction was performed, 100 pg of linearized pGEMEX-1 plasmid RNA (Promega) was added to each fraction to be used as a standard measure.

### qRT-PCR

For qRT-PCRs, polysomal fractions 7–14 for all samples were used individually for gene expression analysis. RNA was resuspended in 15 μl RNase-free H_2_O and 7.5 μl from each fraction was used as a template for cDNA synthesis using M-MuLV reverse transcriptase (NEB) following recommended conditions in 10 μl final volume. After first-strand synthesis, the cDNA was diluted with 30 μl H_2_O, and 2 μl of diluted cDNA were used as a template for real-time qRT-PCR using Ssofast EvaGreen Supermix (Bio-Rad) following the manufacturer’s protocol.

### Library preparation and sequencing

Ribosome profiling was performed using the TruSeq Ribo Profile (Mammalian) Library Prep Kit (Illumina #RPHMR12126) according to the manufacturer’s protocol. The Illumina Ribo-Zero Gold rRNA Removal Kit (H/M/R) (Illumina #MRZG12324) was employed to deplete ribosomal RNA samples. The library quality was verified using a Bioanalyzer. Libraries were sequenced using the NextSeq™ 500 High Output Kit (FC-404-1005) on a NextSeq 500 platform (Illumina) in a 75-bp single read run.

### Data preprocessing and sequence alignment

For 3’ adapter removal, we used the FastX toolkit from the Hannon Lab (http://hannonlab.cshl.edu/fastx_toolkit/). To remove reads originating from rRNA and tRNAs, we aligned the sequences to rRNA and tRNA sequences downloaded from the UCSC genome browser using Bowtie [12] with the following settings: -n 2 -l 20 --best allowing up to 2 mismatches for rRNA and -v 3 -l 20 for tRNAs. The remaining sequences were aligned to the human genome assembly GRCh38/hg38 using Tophat [13] with --bowtie1 option.

### Differential expression and differential translation efficiency analysis

Data analysis was performed using ‘R’ (R Foundation for Statistical Computing, Vienna, Austria), mainly through packages in the Bioconductor suite [14, 15] or in-house-developed scripts. Counts for exons and cds by gene were performed through the GenomicFeatures [16] and the systemPipeR [17] packages using the summarizeOverlaps function with Union mode. Differential expression analysis was performed with edgeR [18, 19]. Only genes with at least 1 count per million total counts in the three biological replicates were considered for the analysis. Differential translation efficiency analysis was performed following the protocol detailed in [20].

### Pathway and gene ontology enrichment analysis

Pathway and Gene Ontology analysis was performed using the clusterProfiler [21], pathview [22], and org.Hs.eg.db packages.

### Motif search and microRNA signatures

To identify conserved motifs in the sequences, we used MEME online suit [23] version 4.12.0. The parameters were set as motif with a minimal width of 6, motif with a maximal width of 20, maximal number of motifs of 10, and ‘zero or one per sequence’. MicroRNA signatures were analyzed using the miREM web analysis tool [24] with default parameters and selecting the following options: species Human, option 2 intersecting two or more databases dynamically, and including non-conserved miRNAs.

### Data set availability

Deep sequencing data from RNA-Seq and ribosome profiling were deposited in the SRA database (https://www.ncbi.nlm.nih.gov/sra/) under accession number PRJNA499096.

### Metabolomics

Metabolites were analyzed by liquid chromatography (LC)-mass spectrometry (MS) (LC-MS/MS) as described [25, 26].

### Western blots

Western blots were performed as previously described [9, 10] using the primary antibodies against MKL1 (ab14984) from Abcam, ERα (sc-543), and p-ERK (sc-7383) from Santa Cruz Biotechnology, ERK 1/2 (4695) from Cell signaling technology, and p-mTOR (5536) from Cell Signaling Technology.

### Immunofluorescence

Cells were grown on 10-mm diameter coverslips in 24-well plates. Cells were fixed with phosphate-buffered saline (PBS) containing 4% paraformaldehyde (PAF) for 10 min and then permeabilized in PBS containing 0.3% Triton X-100 for 10 min. Incubation with the primary antibody (1/1000) was performed overnight (ON) at 4 °C. Primary antibodies against ERα (HC-20, sc-543) and HIF1α (clone 54/HIF1α, 610958) were purchased from Santa Cruz Biotechnology and BD Bioscience respectively. Dye-conjugated secondary antibodies (Abcam) were incubated 1 h at room temperature. The cover slides were mounted in Duolink II mounting medium with DAPI (Sigma-Aldrich), and images were obtained with an ApoTome Axio Z1 Imager microscope (Zeiss).

### 3D matrigel assays

Five thousand cells were plated in medium in a well of a 96-well plate with round bottom previously coated with Poly(2-hydroxyethyl methacrylate) (Sigma) and incubated for 4 days to allow spheroid formation. Matrigel solution was prepared in culture medium at a final concentration of 1 mg/ml. Taken up in Matrigel solution, spheroids were then seeded on the top of a matrigel cushion already formed in 96-well plates. Images were taken by microscopy (DMIRB-Leica).

## Results

### Gene expression patterns and translational efficiency

To understand the main adaptive changes that take place in MCF7-derived cells during the MKL1-induced EM-like transition, we first assessed changes in gene expression at both transcriptional and translational levels. For this purpose, we analyzed the three cell lines (MKL1 ΔN200, MKL1 ΔC301, and MCF7 control cell) after 48 h incubation with tetracycline. We performed deep sequencing of the total RNA as representative of transcriptional gene expression [22]. For the analysis of translation, ribosome profiling was carried out on ribosome-protected fragments (RPFs); these fragments were sequenced and their cumulative values per mRNA are informative of translational expression levels [27]. After quality assessment, tRNAs and rRNAs-originating reads were removed. Reads were next aligned to the genome (Supplementary Table T1). As expected, most of the RPF reads mapped to coding sequences (CDSs), while most of the total RNA reads mapped to both UTRs and CDSs (Supplementary Figure S1). Reads were normalized using the TMM (trimmed mean of M values) normalization method which is a simple and effective method for estimating relative RNA production levels from RNA-seq data [28]. As a cutoff, we set one count per million as a minimum value for an expressed gene and further analyzed only those genes detected over this threshold in three independent biological replicates (Supplementary Table T2 and Supplementary Figures S2 and S3). At the transcriptional level, MKL1 ΔC301 and MCF7 controls expressed a similar number of transcripts, whereas MKL1 ΔN200 cells presented a smaller expression set (86% compared with the MCF7 control). For all three cell lines, the most highly transcribed genes were noncoding, including the signal recognition particle RNA genes (RN7), the RNA component of the RNase P ribonucleoprotein (H1RNA), and several small nuclear RNA genes.

**Fig. 1:**
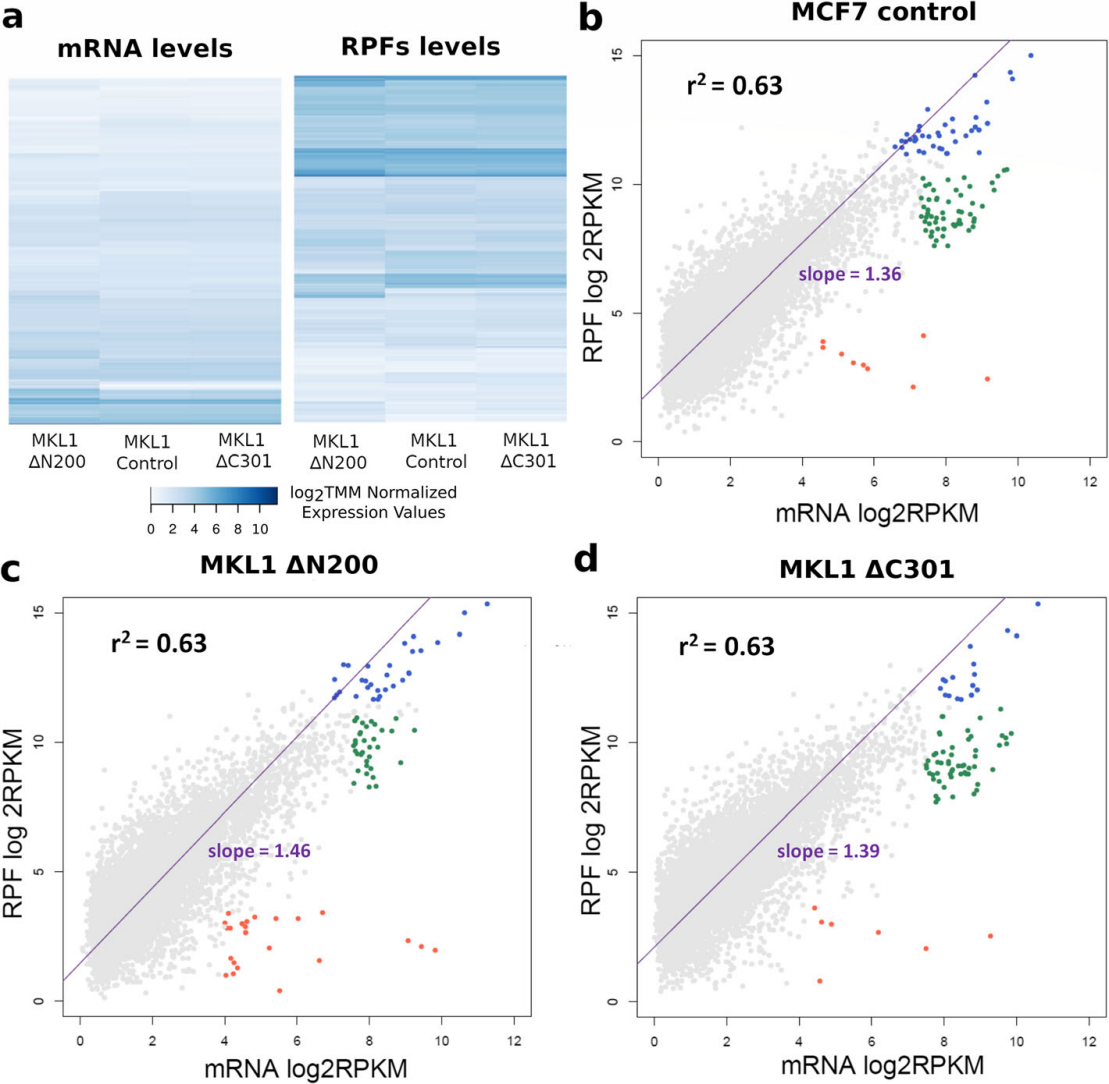
Genes expressed in the cell lines. **a** Heatmaps show global expression patterns. Left: heatmap representing mRNA levels. Right: heatmap representing RPF levels. Each column represents a cell line and each row an expressed gene. log2TMM-normalized values are plotted. The color code represents expression data levels: light shades of blue indicate low expression levels, while strong shades indicate high expression levels. **b** The biplot shows the log2 RPKM of RPFs (y-axis) and mRNA (x-axis) of genes expressed in MCF7 control, **c** MCF7 MKL1 ΔN200, and **d** MCF7 MKL1 ΔC301. The coefficient of determination is shown in black while the linear regression slopes are shown in violet. Gene groups are depicted in different colors: in red, genes with high transcription and low translation; in blue, genes with high transcription and very high translation; in green, genes with high transcription and translation. TMM, trimmed mean of M values; RPKM, reads per kilobase million

MKL1 ΔC301 and MCF7 control cells seemed to be the most alike, whereas MKL1 ΔN200 cells were the most divergent (Fig. 1a). Among the three cell lines, we observed a moderate general correlation between transcriptional and translational programs (Fig. 1b-d). Thereby, genes with high total RNA counts also had a high number of RPFs, suggesting a general expression coordination. Compared with MKL1 ΔC301 and MCF7 control cells, MKL1 ΔN200 cells exhibited a higher translational efficiency (95% confidence intervals for the slopes [1.337, 1.383], [1.434, 1.486], and [1.370, 1.415] for MCF7 control, MKL1 ΔN200 and MKL1 ΔC301, respectively).

The expression profiles were separated into three distinct groups (Supplementary File S1). Most genes with high total RNA and RPF counts (Fig. 1b–d, green dots) corresponded to ribosomal proteins and translation elongation factors. Genes with high total RNA and very high RPF counts (Fig. 1b–d, blue dots) included keratins and genes associated with the cytoskeleton. Additionally, a number of histone-coding genes were detected in this group in the control cells. The subset of genes with high total RNA and very low RPFs counts were present mainly in MKL1 ΔN200 (Fig. 1b–d, red dots), and they seemed to be associated with unrelated functions.

In summary, the three cell lines shared expression patterns with cell-type-specific features. All cell lines showed a moderate correlation between total RNA and RPFs counts suggesting a correlation between transcriptional and translational programs. Genes corresponding to ribosomal proteins and elongation factors exhibited high expression at both levels. In addition, MKL1 ΔN200 was the most divergent cell type, showing a slightly decreased repertoire of active genes with higher translation efficiency.

### Differentially expressed genes validate the induction of an EM-like transition in MKL1 ΔN200 cells

**Fig. 2:**
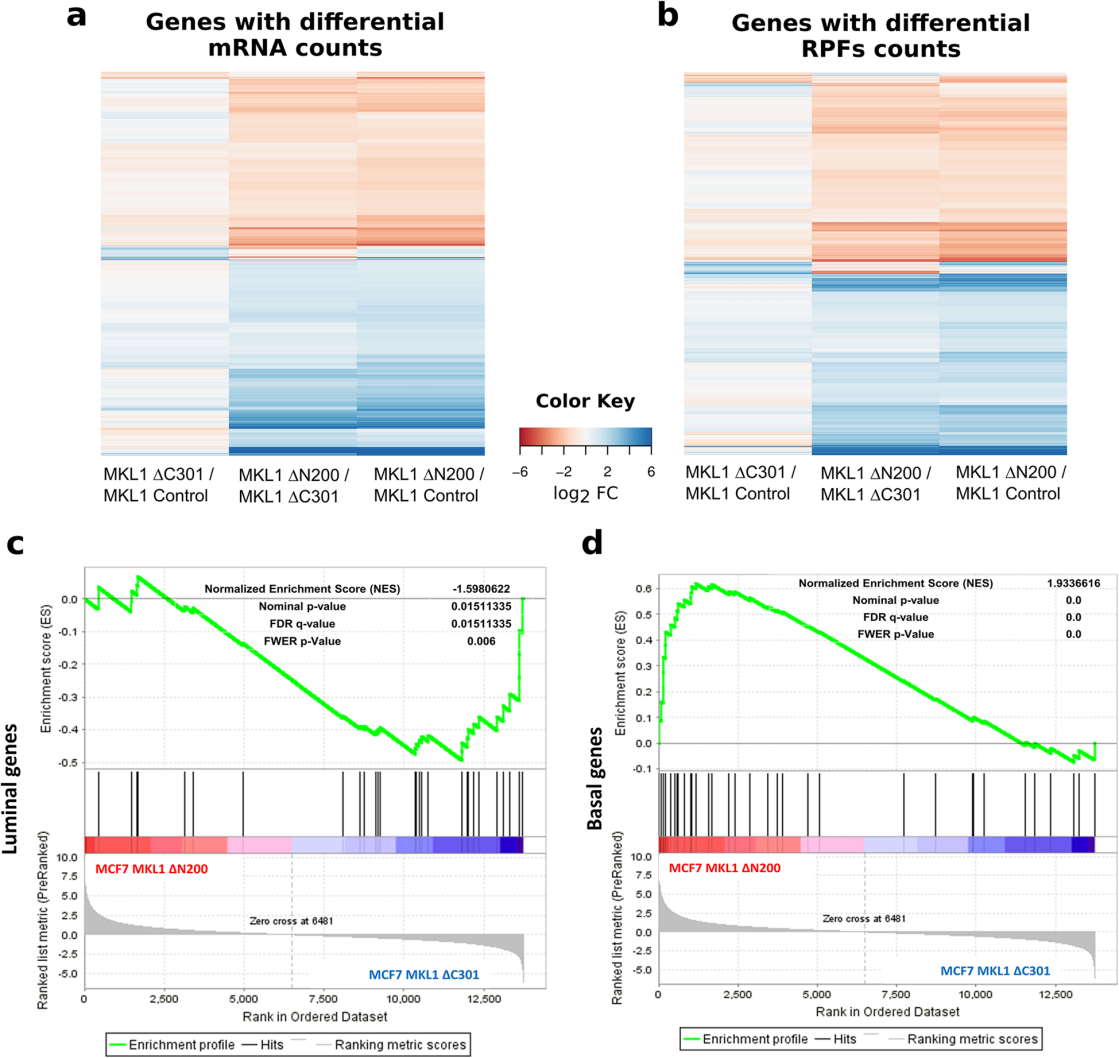
Differential gene expression at transcriptional and translational levels reveal a basal-like gene expression signature in MCF7 MKL1 ΔN200 cells. Heatmaps showing log2 TMM fold changes of genes with differential mRNA counts (**a**) and differential RPFs counts (**b**) genes between the cell lines. The color code represents fold changes levels: shades of red indicate a diminished expression while shades of blue indicate overexpression. **c** Gene set enrichment analysis (GSEA) showing the association of differentially expressed genes to luminal and **d** basal marker gene sets. The bar-code plot indicates the position of the genes on the expression data rank-sorted, with red and blue colors indicating over- and underexpression in MCF7 MKL1 ΔN200 compared to MCF7 MKL1 ΔC301 control cells, respectively. The enrichment plot for basal genes is skewed to the left, indicating an association of MCF7 MKL1 ΔN200 to the basal markers gene set. The enrichment plot for luminal genes is skewed to the right, indicating an association of MCF7 MKL1 ΔC301 control cells to the luminal marker gene set. Significance statistics for GSEA is shown on top of the gene set enrichment plot. TMM, trimmed mean of M values

To characterize the transition undertaken by MKL1 ΔN200 compared with MKL1 ΔC301 and MCF7 control cells, we performed a differential gene expression analysis among the three cell lines. For both RNA and RPF sets, we defined a list of differentially expressed genes setting an arbitrary cutoff of a Benjamini FDR adjusted *p* value < 0.01 and a fold change > ± 2 (Fig. 2a–b and Supplementary Files S2 and S3). With these criteria, we found more than 3.000 differentially expressed genes between MKL1 ΔN200 and either MKL1 ΔC301 or MCF7 control in both total RNA and RPF analysis. Notably, MKL1 ΔC301 and MCF7 control cells showed less than 500 differentially expressed genes (Supplementary Files S2 and S3). To test data consistency, we compared log2 fold changes from total RNA differential expression analysis to log2 fold changes from microarray data recently published [29]. We found a significant correlation between both data sets (correlation coefficient 0.84, *p* < 2.2 e−16).

The ontology analysis of gene expression between MKL1 ΔN200 and control cells revealed an enrichment of those involved in the regulation of cell adhesion, actin cytoskeleton organization, and migration (Supplementary Files S5, S6 and S7). Indeed, all six family members of the actin genes (ACTN) and FLNA, which is an actin-binding protein that crosslinks actin filaments, are overexpressed. Similarly, a number of genes involved in kinase cascades and signal transduction are upregulated, consistent with the induction of an EM-like transition [30]. We also found an underrepresentation of genes involved in epigenetic marks such as chromatin remodeling and DNA methylation, which indicates cell cycle arrest, consistent with the induction of an EM-like transition (reviewed in [29, 30]).

We then analyzed changes in total mRNA and RPF levels of some EMT markers (Supplementary Files S2 and S3). As expected, for several epithelial markers like EPCAM, KRT8, KRT18, TJP3, and GATA3, expression levels were reduced in the MKL1 ΔN200 cells compared to control cell lines. Similarly, gene expression changes associated with breast EMT and cancer progression were detected, as the reduction of ERa and ERBB2 [29] and the increase of NOTCH1 and WNT5B [31, 32]. In contrast, no decrease in E-cadherin levels was observed in MKL1 ΔN200. This is consistent with previous observations that show the disassembly of E-cadherin fibers [9]. The expression of mesenchymal markers associated with cancer progression [30], such as FN1, VTN, and ITGA5, was markedly increased (between 20- and 50-fold). We then checked if the expression profile of the EM-like transition also presents traits of a luminal to basal change as previously reported [9]. Gene set enrichment analysis revealed a significant association of MCF7 control and MKL1 ΔN200 cells to the luminal (Fig. 2c) and to the basal (Fig. 2d) markers gene set, respectively. As a SRF cofactor we wondered how a constitutively active variant of MKL1 could affect SRF and its targets. Results showed that SRF was overexpressed as well as several SRF target genes with some of them being more than 100-fold overexpressed (TAGLN and several myosin light chains).

To confirm some of these findings, we first analyzed by western blots the expression of specific markers after tetracycline-induced production of MKL1 ΔN200 and MKL1 ΔC301 mutants in MCF7 cells. The expression of the tagged-MKL1 variants appeared 24 h after tetracycline treatment. As expected [9], expression of MKL1 ΔN200 variant clearly induced the expression of alpha actin and downregulated ERa expression, indicating the implementation of a dedifferentiation process of the luminal cells (Supplementary Figure S4). Increase in ERK phosphorylation status in these cells further confirmed an activation of the MAPK/ERK signaling pathway as suggested by GO analysis (Supplementary File S5 and S7). Furthermore, the motile invasion properties of MCF7 MKL1 ΔN200 cells suggested by GO analysis (Supplementary File S5 and S7), were illustrated by a 3D spheroid invasion assay on Matrigel. Only MCF7 cells expressing MKL1 ΔN200 variant showed invasive capability of the Matrigel (Supplementary Figure S5) which was previously quantified [29]. Interestingly, these cells also increased their size but decreased their viability after MKL1 ΔN200 expression with tetracycline. After 48 h of induction, MKL1 ΔN200 cells reached around 60% viability, whereas the control cell lines remained mostly constant (Supplementary Figure S6).

In sum, our results confirmed that after 48 h incubation with tetracycline, MKL1 ΔN200 and MKL1 ΔC301 truncated proteins are highly expressed in the corresponding cell lines (Supplementary Figure S4). Moreover, in these experimental conditions, we verified that MKL1 ΔN200 phenotype resembles an EMT state in which the expression of factors that promote mesenchymal transition is upregulated in contrast to the reduced expression of several epithelial markers.

### During the EM-like transition, various pathways are specifically regulated at the transcriptional or translational level

**Fig. 3:**
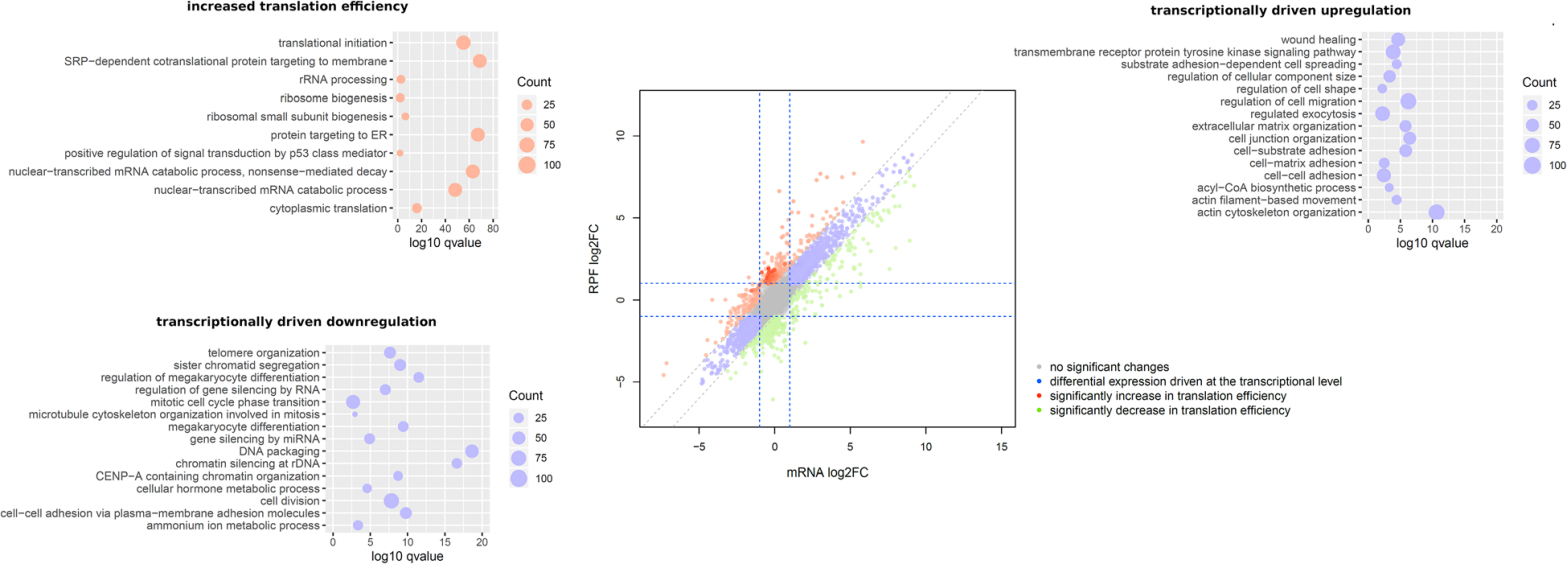
Some pathways are specifically regulated at transcriptional or translational levels. Biplot showing the log2-fold TMM differences of RPFs (y-axis) and mRNA (x-axis) between MCF7 MKL1 ΔN200 and MCF7 MKL1 ΔC301. Genes with expression changes driven by transcription regulation are shown in blue, genes with increased translation efficiency in red, and genes with decreased translation efficiency in green. Color shades represent log10 *p* values resulting from the differential translation efficiency analysis: light shades indicate high values while strong shades indicate low values. Genes were considered differentially expressed if *p* value < 0.01 and abs(FC) > 2. The fold change cutoff value is indicated as a dashed line. Summary of the GO term enrichment analysis performed with the different group of genes between MCF7 MKL1 ΔN200 and MCF7 MKL1 ΔC301 control is shown. Selected GO classes with an overrepresentation are indicated. For genes with expression changes driven by transcription regulation upregulated and downregulated genes were used independently in the GO analysis. TMM, trimmed mean of M values

To assess whether differentially expressed genes are mainly regulated transcriptionally or translationally, we compared total RNA and RPFs counts per transcript between cell lines (Fig. 3). We reasoned that if the regulation of gene expression is mostly driven at the transcriptional level, an increase or reduction in the RNA reads should be paralleled by the gain or loss of the RPF reads. Alternatively, if the regulation of gene expression is executed at the translational level, changes in the RPF reads should not be accompanied by changes in the RNA reads to the same extent. That is, any significantly off-diagonal point when comparing RNA to RPFs represents a gene with decoupled regulation between transcription and translation.

One-third of the genes presented decoupled regulation of transcription and translation. To explore which pathways were specifically regulated at the transcriptional or translational level, we defined three groups of genes as inputs for ontology analysis: (i) genes with significant changes in both mRNA and RPFs counts but with mRNA log2 FC equal to RPFs log2 FC ±1 (Fig. 3 and Supplementary Figures S7 and S8, blue dots); (ii) genes with RPF log2 FC > mRNA log2 FC ± 1 which represent genes with increased translation per mRNA or increased translation efficiency (Fig. 3 and Supplementary Figures S7 and S8, red dots), and (iii) genes with RPF log2 FC < mRNA log2 FC ± 1 which represent genes with decreased translation efficiency (Fig. 3 and Supplementary Figures S7 and S8, green dots). Group (i) represents genes which are transcriptionally regulated, while groups (ii) and (iii) represent genes with decoupled regulation between transcription and translation. In addition, we performed a differential translation efficiency analysis which was consistent with the defined groups (Supplementary File S4). Figure 3 summarizes the enriched GO terms in MKL1 ΔN200 cells compared with the MKL1 ΔC301 control cells (the complete list is in Supplementary File S5). Between MKL1 ΔN200 and MKL1 ΔC301 control cells, the ontology analysis of group (i) revealed, as expected, a high representation of genes involved in an EM-like transition state. Strikingly, the GO terms of the genes from group (ii) showed an overrepresentation of genes related to translation machinery. Among them, translation was increased for genes involved in ribosome biogenesis, ribosome assembly, initiation factors, and other general cytoplasmic translation factors. Comparisons between MKL1 ΔN200 and MCF7 control cells and between MKL1 ΔC301 and MCF7 control cells are presented in Supplementary Files S6 and S7 and Supplementary Figures S7 and S8, respectively.

To explore if particular miRNA or ribosome-binding proteins could be involved in some of the post-transcriptional regulatory mechanisms, we searched for common microRNA signatures and 5’ or 3’ UTR motifs in groups (ii) and (iii). We found that miR-520 family appears to play a role in the regulation of these groups of genes (Supplementary Figure S9). Among the 664 genes, 269 were targets of hsa-mir-520d-3p. Meanwhile when analyzing those with decreased translation efficiency only (group (iii)) other microRNA appear to be relevant as well (Supplementary Figure S10). For instance, 190 of the 338 downregulated genes were targets of hsa-mir-661. On the other hand, motif analysis showed that subsets of these genes shared sequence motifs on their UTRs. One of these motifs is an ELAVL1 binding site, a ribosome-binding protein previously involved in translational regulation of EMT [33–35]. Hence, the combination of ribosome profiling with RNA-Seq suggests that cells employ different strategies for regulating gene expression and expose different regulatory programs in the induced EM-like transition.

### In MKL1 ΔN200, the higher expression of translational machinery components is regulated at the translational level

**Fig. 4:**
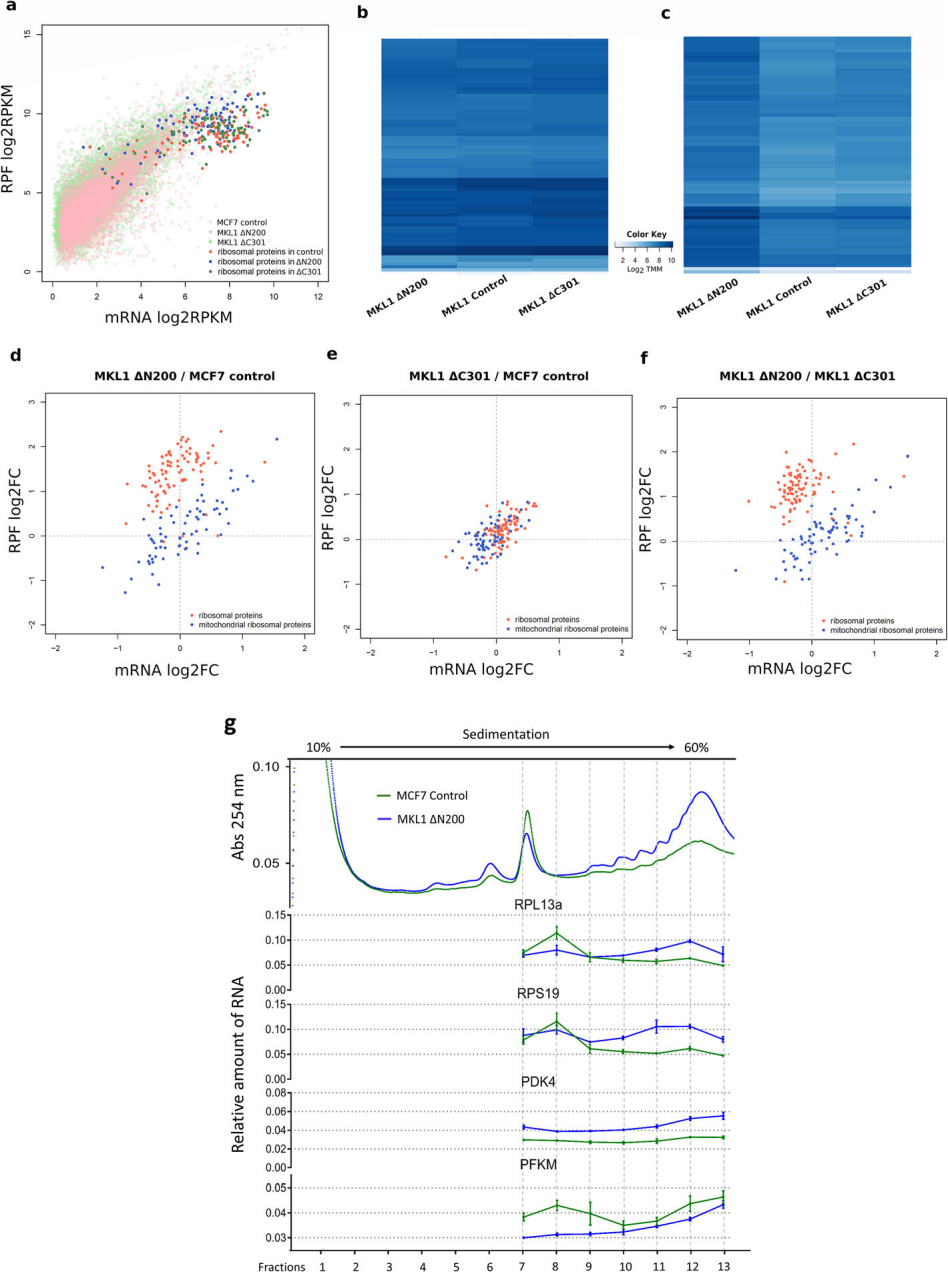
Characterization of ribosomal proteins expression. **a** Biplot showing the log2 RPKM of RPFs (y-axis) and mRNA (x-axis) of genes. The expression of ribosomal proteins is highlighted in bright colors. Heatmaps of the log2 TMM of mRNA (**b**) and RPFs (**c**) of ribosomal protein genes, respectively. The color code represents expression data levels: light shades of blue indicate low expression levels (low log2 TMM values) while strong shades indicate high expression levels (high log2 TMM values). Biplots showing the log2-fold RPKM differences in RPFs (y-axis) and mRNA (x-axis) in ribosomal protein expression between MKL1 ΔN200 and MCF7 control (**d**), MKL1 ΔC301 and MCF7 control (**e**), and MKL1 ΔN200 and MKL1 ΔC301 (**f**) respectively. **g** Polysome profiles of MCF7 control and MKL1 ΔN200. Polysomal fractionation was done as described [15] with some modifications. Relative amount of RNA values refers to the inverse of the corrected Cq values (corrected Cq^−1^) from qRT-PCRs experiments. Error bars represent standard deviation from experimental triplicate measurements. RPKM, reads per kilobase million; TMM, trimmed mean of M values

The general increase in translation of transcripts associated with the translation machinery encouraged us to further characterize their expression. A similar global expression pattern of all 88 cytosolic ribosomal proteins was observed for the three cell lines (Fig. 4a–c). However, comparison with the MCF7 control and MKL1 ΔC301 cells revealed an increase in RPFs in MKL1 ΔN200 cells (Fig. 4a; shift to the top of the blue spots). Differential expression analysis showed that in MKL1 ΔN200 cells, the 88 cytosolic ribosomal proteins exhibited significantly higher RPF reads without significant changes in their mRNA expression compared to control cells (Supplementary File S8, Fig. 4d, f). In contrast, the mitochondrial ribosomal proteins remained unchanged among all three cell lines. Most cytosolic ribosomal proteins in MKL1 ΔN200 cells were upregulated solely at the translational level (69 and 62 out of 88 compared with the MCF7 control and MKL1 ΔC301 cells, respectively). Only RPS26 was upregulated at both levels (Supplementary File S8). RPS27A and RPL9 exhibited the highest expression in MKL1 ΔN200 cells (5-fold).

Unlike the uniform translational regulation of cytosolic ribosomal proteins, the expression of initiation translation factors and associated molecules seems to be regulated by diverse mechanisms. For some factors, transcription and translation decrease (EIF4A3 and EIF2AK1) or increase (EIF2AK2, EIF1, EIF5, EIF6), whereas, for others, translational and transcriptional changes obey opposite trends (eIF3 family, EIF4B, and EIF4A2). This particular regulation of translation initiation factors suggests a selective regulation of gene expression at the translational level. In MKL1 ΔN200, the translation of all cytosolic elongation factors was upregulated, which seemed to contribute to the general increase in translation in these cells. It has been reported that the expression of ribosomal proteins and elongation factors can be regulated translationally through TOP sequence in the 5’ UTR of their mRNAs [36, 37]. To explore if this could be the case in MKL1 ΔN200 cells, we used genes with differential translation efficiency (Supplementary File S4) to perform a gene set enrichment analysis. Our results revealed a significant association of MKL1 ΔN200 cells with the translation efficiency of the 5’TOP containing genes (Supplementary Figure S11). This result suggests that 5’TOP sequences likely regulate translation of the translation machinery components in MKL1 ΔN200 cells. However, we cannot propose a mechanism. mTORC pathways were involved in regulation of 5’TOP-containing genes [38, 39]. In MKL1 ΔN200 cells, the expression of several molecules associated with mTORC-linked pathways, as well as some components of the two mTOR multiprotein complexes, are affected differently, while TOR expression levels themselves do not change significantly. In addition, La-related protein 1 (LARP1), a key player in ribosomal protein synthesis that controls the stability of the 5’TOP mRNAs [40, 41], shows no significant changes in MKL1 ΔN200 cells compared to control cells.

The expression increase of ribosomal proteins, elongation factors, and others associated with ribosome biogenesis suggested a general translation increase. This was supported by polysome profiles (Fig. 4g, upper panel) which also suggested an increase in translation efficiency. The profile is shifted towards higher fractions in MKL1 ΔN200 compared to control cells indicating there are, on average, more ribosomes per mRNA. To test changes in gene expression, we selected four genes with different transcription and translation behavior and performed RT-qPCRs from each individual fraction of the polysome profile. Genes selected were PDK4 which is upregulated both at transcription and translation, RPS19 which is upregulated only at translation, PFKM downregulated both at transcription and translation, and RPL13A which is downregulated at transcription but upregulated at translation. RT-qPCRs results confirmed the expected changes (Fig. 4g, Supplementary Table T3 and Supplementary Figure S12). Moreover, a right shift of mRNA signal into the higher polysome fractions can be observed for the 4 genes indicating an increase in translational efficiency in all of them (Fig. 4g down panel and Supplementary Table T4).

Taken together, our results showed a clear increase in the synthesis of ribosomal proteins, which was mainly regulated at the translational level during the EM-like transition. We also detected an upregulation of factors related to translation. These results, together with those from polysome fraction analysis, indicate an increased and more efficient translation activity.

### Adaptive changes in cellular metabolism

**Fig. 5:**
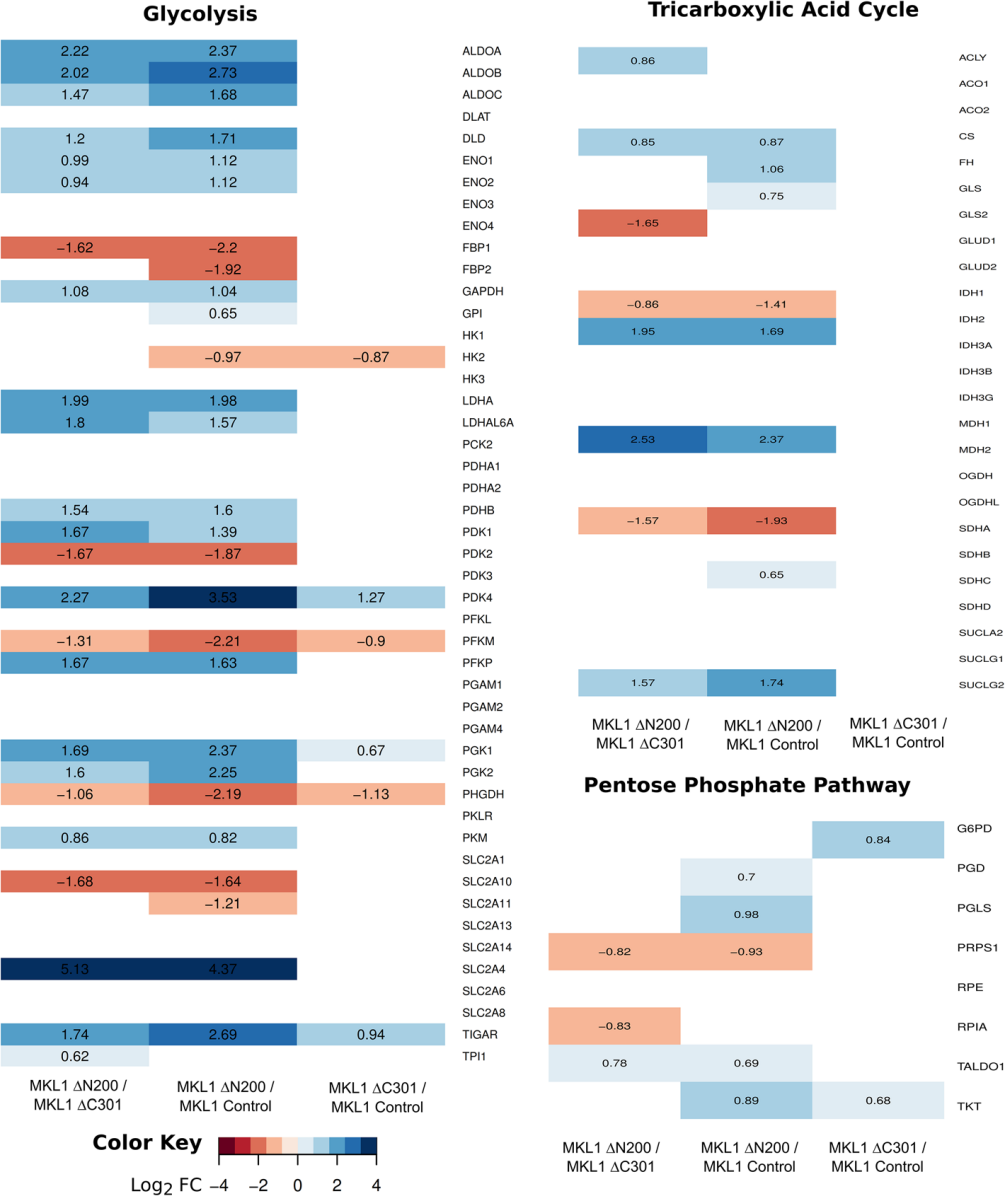
Gene expression analysis of genes participating in glycolysis, tricarboxylic acid cycle, and pentose phosphate pathway. Heatmaps of the log2 TMM fold changes of RPFs of the genes involved in glycolysis, TCA, and PPP. Expression values are shown for genes with FDR < 0.01. The color code represents fold changes levels: shades of red indicate underexpression (negative log2 FC values) while shades of blue indicate overexpression (positive log2 FC values). TMM, trimmed mean of M values

**Fig. 6:**
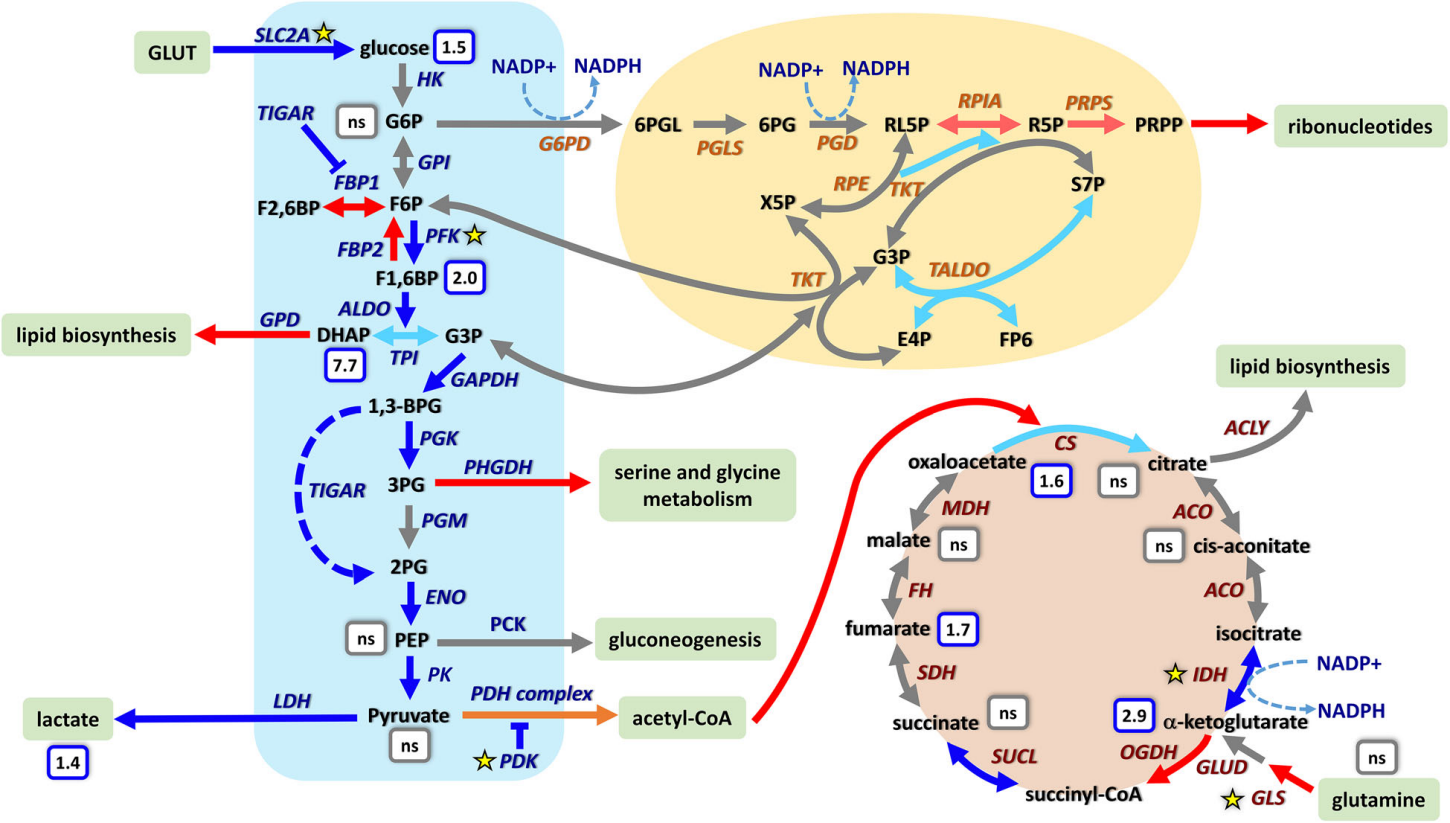
Schematics of glycolysis, tricarboxylic acid cycle, and pentose phosphate pathway showing changes at the translational level of enzymes involved in the different steps. Changes in MCF7 MKL1 ΔN200 with respect to MCF7 MKL1 ΔC301 control cells. Fold-change (FC) values were taken from Supplementary File S3; only FC values higher than 0.5 (FDR < 0.01) were considered. Blue, glycolysis; orange, PPP; pink, TCA. Arrow colors indicate the following: increase (blue: FC > 1.0, light blue: 1.0 > FC > 0.5); decrease (red: FC > 1.0, light red: 1.0 > FC > 0.5); gray: no significant changes; orange: variable changes within an enzyme complex. Values shown inside the small boxes indicate the fold change in the amount of each metabolite between both cell lines (Supplementary Table T6). Stars depict changes in the prevailing isoform. Steps associated with NADP reduction are indicated

Protein biosynthesis and ribosome biogenesis are among the most energy-consuming processes in the cell [42, 43], raising the question as to how cellular metabolism in MKL1 ΔN200 cells adapts during the EM-like transition. The differential expression analysis revealed some metabolic pathways as enriched GO terms (Supplementary Files S5 and S7). Therefore, we next examined closely the expression changes in the enzymes involved in energy metabolism (Fig. 5 and 6). For most enzymes, we detected roughly similar changes in both mRNA and RPFs levels (Supplementary Files S2 and S3). RPFs changes, assembled into the corresponding pathways, are summarized in Fig. 6.

#### Glycolysis

In MKL1 ΔN200 cells, the pathway leading to lactate production showed a general increase in the expression of most enzymes (Figs. 5 and 6). Interestingly, when comparing MKL1 ΔN200 with MKL1 ΔC301 control cells, several steps of the pathway presented marked changes. The expression of glucose transporter SLC2A4 strongly increased (more than 30-fold), whereas that of SLC2A10 and SLC2A11 decreased. PFK-1, the main regulatory enzyme of glycolysis, showed lower expression of its isoform PFKM while that of the low-affinity PFKP isoform significantly increased. The expression levels of FBP1 which is involved in the reverse reaction from fructose-1.6 bisphosphate to fructose-6 phosphate associated with the PFK step, markedly decreased. Finally, the expression of ALDO A, B, and C and both PGK isoforms increased several folds.

Regarding the pathway flow, we observed an increase in the expression of all LDH isoforms responsible for lactate production from pyruvate. This was experimentally validated by both metabolomic analysis (Fig. 6 and Supplementary Table T6) and post-induction lactate measurements in the cell-conditioned media (Supplementary Figure S6b and Supplementary Table T5). We also detected a decrease in the expression of the enzymes responsible for directing the pathway flow towards other metabolic pathways. Our data strongly suggest that the step leading from pyruvate to acetyl-CoA was severely impaired in MKL1 ΔN200 cells. The expression levels of PDH complex components varied. Concomitantly, the expression of their inhibitor (PDK) markedly augmented. Indeed, the expression of the isoforms PDK1 and PDK4 increased, PDK3 remained unchanged while PDK2 decreased compared with control cells. Notably, PDK4 increased 4.8- and 12-fold compared to MKL1 ΔC301 and MCF7 control cells respectively. Additionally, a reduction of the expression of GPD1 and PHGDH was observed, suggesting that the flow towards lipid synthesis from dihydroxyacetone phosphate and diversion towards serine biosynthesis from 3-phosphoglycerate would decrease. Concerning the flow towards gluconeogenesis, no significant changes were detected for cytosolic and mitochondrial PCK. A further change in MKL1 ΔN200 cells linked to glycolysis pathway should be mentioned: the expression of the p53-dependent TIGAR, which increased 6-fold and 3-fold compared with that in MCF7 and MKL1 ΔC301 cells (Figs. 5 and 6).

Comparisons between MKL1 ΔC301 and MCF7 control cells (Supplementary Figure S13a) revealed only few changes in the expression of the enzymes involved in glycolysis. Similar to MKL1 ΔN200 cells, MKL1 ΔC301 cells exhibited an increase in the expression of PDK4, albeit to a much lesser extent than MKL1 ΔN200 cells (2.4-fold). Finally, the expression of enzymes that redirect the metabolic flux towards serine metabolism also decreased.

In summary, gene expression analysis suggested that in MKL1 ΔN200, glycolysis was fully active, with a marked increase in glucose uptake and lactate production. This was confirmed experimentally by detecting a significant change in the glucose consumption/lactate production rate (Supplementary Table T5 and Supplementary Figure S6b) and by global metabolomic measurements that showed an intracellular increase of glucose and F1,6BP (Fig. 6 and Supplementary Table T6). Gene expression analysis also revealed changes in the expression of critical isoforms responsible for substrate uptake and pathway regulation. The subsequent feeding into the TCA cycle would be decreased as well as the diversion towards lipid biosynthesis and serine metabolism.

#### Tricarboxylic acid cycle

As shown in Figs. 5 and 6, the expression of enzymes involved in the TCA cycle was strongly altered in MKL1 ΔN200 cells compared with that in the MKL1 ΔC301 cells, whereas it was similar between MKL1 ΔC301 and MCF7 control cells (Fig. 5 and Supplementary Figure S13a). The main expression changes in MKL1 ΔN200 cells were as follows: a significant increase in PDK, the PDH complex inhibitor, an increase in CS and IDH2, and a decrease in the decarboxylating component of OGDH. Concerning the cycle feeding from glutamine, a decrease of GLS2 isoform expression was observed. Furthermore, expression of the cytoplasmic MDH1, involved in the malate-aspartate shuttle, increased roughly 6-fold. In summary, gene expression analysis suggested an alteration of the TCA cycle in MKL1 ΔN200 cells which is consistent with the metabolomic measurements (Fig. 6).

#### Pentose phosphate pathway (PPP)

In MKL1 ΔN200 compared with MKL1 ΔC301 control cells, the oxidative phase presented a decreased expression of RPIA and PRPS enzymes (Figs. 5 and 6), and the nonoxidative phase showed an increased TALDO expression. In MKL1 ΔC301 compared with MCF7 control cells (Fig. 5 and Supplementary Figure S13a), only G6PD and TKT increased. In summary, our results suggest that PPP flow is oriented towards maintaining the redox status through NADPH production without ribonucleotide synthesis and towards metabolite recycling and supply.

#### HIF-1α control

HIF-1α is a major transcriptional regulator involved in the Warburg effect. In MKL1 ΔN200 cells, the expression of HIF-1α and HIF-2α (EPAS1 gene) was significantly increased, whereas the expression of E3-ligase VHL (Von Hippel-Lindau factor), implicated in HIF-α ubiquitination for proteasomal degradation under normoxic conditions, was decreased. A further level of HIF-1α control recently described involves two long noncoding RNAs, HIF1A antisense RNA1 (HIF1A-AS1) and HIF1A antisense RNA2 (HIF1A-AS2), in a yet unclear mechanism [51]. Notably, the transcription of both antisense RNAs was clearly increased in MKL1 ΔN200 cells. The significant HIF-1α expression increase in MKL1 ΔN200 cells, indicated by mRNA and RPFs levels, is consistent with the augmented nuclear location revealed by in situ immunofluorescence (Supplementary Figure S14).

In summary, our results provide strong evidence for a deep metabolic adaptation of MKL1 ΔN200 cells, which involves fully active glycolysis, severely perturbed TCA cycle and an active PPP with decreased ribonucleotide production. This metabolic rewiring is likely driven by HIF-1α that presents increased level and nuclear location

## Discussion

During breast cancer progression, the underlying mechanisms of metastasis and hormonal therapy resistance are still elusive; however, they have been associated with cellular changes that occur in EMT. To gain detailed insights into breast cancer progression, here, we characterized the adaptive changes that occur during the MKL1-induced EM-like transition employing a MCF7-derived cellular model. To analyze our results, we cannot skip mentioning some aspects of the cellular model: (i) the viability decrease of EMT-undergoing cells after long culture periods, (ii) the intrinsic heterogeneity of cellular models that makes the results actually averages. (iii) Our work analyzed just the final state after 48 h of the induction of MKL1 variants expression. However, the results we present here supported by the use of two different control cells, together with the studies reported in Jehanno and Fernández-Calero et al. [29], allow us to validate the current cell model to deepen studies on EMT processes.

Unlike MKL1 ΔC301 and MCF7 control cells, in MKL1 ΔN200 cells, actins and FLNA are overexpressed. Interestingly, FLNA has been proposed to be a regulator of nuclear actin polymerization and, hence, of SRF target gene expression. Indeed, the effect of MKL1 ΔN200 seems to be mediated by SRF, at least in part, since SRF is overexpressed as well as several SRF target genes (actin, TGLN, CCN1, CCN2, and several myosins). Of note, CSRP2, which is an invadopodia actin-bundling protein that is upregulated by hypoxia (HIF-1α) in various breast cancer cell lines and tumors, is also upregulated in MKL1 ΔN200 cells. Together, the expression of MKL1 ΔN200 led to upregulation of beta actin and related proteins involved in actin dynamics, cytoskeleton, intercellular signaling, cell shape, and locomotion.

Among breast cancer subtypes, luminal tumors appear to have cells solely on the epithelial edge of the EMT spectrum while basal-like tumors are more heterogeneous with cells spanning the spectrum from potential progenitors to mesenchymal-oriented variants [6]. Accordingly, MKL1 ΔN200 cells have been previously described to have a typical profile of triple-negative breast cancer or basal-like tumors [9]. Our results confirmed that MKL1 ΔN200 cells have a basal-like expression profile while both control cells have a luminal one. With respect to EMT markers, although translation of the epithelial markers CDH1 and CLDN1 is increased in MKL1 ΔN200, others such as EPCAM, KRT8, KRT18, TJP3 and GATA3 are reduced. In contrast, expression of the mesenchymal markers VIM and CDH2 is not altered, but other markers such as FN1, VTN, and ITGA5 are highly increased. Moreover, the expression of the EMT mediators Notch1 and Wnt5B is increased, both of which are associated with breast EMT and cancer progression [44–46]. Furthermore, in association with endocrine therapy resistance, the expression of two hormonal receptors, ESR1 and ERBB2, is strongly reduced in MKL1 ΔN200 cells.

SRF is involved in cellular reprogramming and is activated by a variety of extracellular signals and, in different cell types, can destabilize cell identity in response to diverse signals [47]. The overexpression of SRF in MKL1 ΔN200 cells may contribute to MCF7 dedifferentiation, leading to more immature cellular traits, which is consistent with an EM-like transition and the adoption of cancer stem cell features. In fact, MKL1 ΔN200 cells show an increase in the expression of CD44 and a decrease in CD24 compared with the control. CD44 is overexpressed in breast tumor cells [48], and the ratio of CD44 to CD24 has been used as a marker for stem cells in breast cancer. This ratio increases in MKL1 ΔN200 cells. Notably, ALDH1 expression, another marker of cancer stem cells [49], is not differentially expressed. Taken together, these results suggest that the MKL1 ΔN200 phenotype corresponds to an EMT state with immature cellular traits, in which the expression of several epithelial markers together with two endocrine receptors involved in the hormonal response is reduced, whereas that of several mesenchymal markers is increased. This interpretation is consistent with a recent study indicating a high flexibility in this transitional process, in which cells no longer oscillate between full epithelial and defined mesenchymal states but rather sample a spectrum of intermediary states [50].

Our results exposed different regulatory programs in the induced EM-like transition process. On one hand, changes in the expression of widely known EMT-related pathways are mostly transcriptionally regulated while, on the other hand, expression changes of other pathways are translationally regulated. Gene expression regulation through translation has been previously described to play a critical role in EMT induction, including a switch from cap-dependent to cap-independent translation [51–53]. However, some of the genes that play key roles in the induction of EMT can be translationally controlled by RNA-binding proteins like ELAVL1, LARP1, and PCBP1 suggesting far more complex regulation of the EM-like transitions. In our model, regulation is not solely dependent on the switch between cap-dependent and cap-independent translation. In fact, our results suggest an interplay of different regulatory strategies (including miRNA, ribosome-binding proteins as well as the expression of particular initiation factors) working together to shape the translation landscape in the induced EM-like transition. Our results also suggest that miRNA-520 family could play a role in defining this landscape, elements previously described as involved in post-transcriptional regulation in breast tumors [54, 55].

Interestingly, the synthesis of the translation apparatus itself is regulated at the translational level in MKL1 ΔN200 cells. Ribosomal protein expression in higher eukaryotes is regulated translationally through a TOP sequence in the 5’ UTR of their mRNAs [36, 37] by a mechanism that involves the mTORC pathway [38, 39]. Our results show a significant increase in translation efficiency of all the genes which have been confirmed to be regulated through 5’TOP sequences. The observed increase in translation of ribosomal proteins and elongation factors, together with the previously described 2-fold augmentation in RNA [10], point towards an increased ribosome biogenesis, consistent with a recent report [56], suggesting that ribosome biogenesis is a general feature of the EM-like transition programs. Furthermore, the significant increase in the translation efficiency and protein biosynthesis in MKL1 ΔN200 cells correlates with their larger cell size (Supplementary Figure S6c) and total protein content [10].

In MKL1 ΔN200 cells, the expression of enzymes related to the TCA, glycolysis, pentose phosphate pathways, and connected metabolic processes broadly agree with previous descriptions for metastatic cells and the EMT [57–59]. The general scheme appears to be consistent with a Warburg-like effect, where HIF-1α should play a master regulatory role in the metabolic changes [60, 61]. These changes include impairment of TCA, increased glucose consumption and glycolysis, and expression changes in enzymes/isozymes involved in energy metabolism. It should be emphasized that changes in MKL1 ΔN200 cells occur in a pseudohypoxia context, and a Warburg-like effect in which the expression of HIF-1α is already enhanced. In this regard, it is important to highlight changes in MKL1 ΔN200 linked to HIF1-α biosynthesis, stability, and nuclear localization; on the other hand, changes in its metabolic targets.

Alterations in the expression of TCA enzymes suggest interesting clues related to triggering an “enhanced pseudohypoxia state”. In MKL1 ΔN200, the main changes in TCA enzymes are on *α*-ketoglutarate (*α*-KG) and succinate. Studies carried on MCF7 cells revealed a general decrease respective to control MCF10 cells in the biosynthesis of TCA enzymes (except for MHD2, reported as roughly constant), a marked increase in IDH3, and a decrease in IDH2 expression [68]. In MKL1 ΔN200, a critical isoform switch from mitochondrial IDH3 to IDH2 is apparent. Cytosolic IDH1 and mitochondrial IDH2 are homodimers that reversibly catalyze the decarboxylation of isocitrate to *α*-KG, whereas IDH3 is a heterotetramer that only oxidizes isocitrate [46]. This phenomenon strongly suggests that α-KG should play a critical role in the induced cellular transition. Indeed, while the decrease in OGDH expression suggests an accumulation of α-KG (confirmed by metabolomic analysis), the reductive carboxylation allowed by IDH2 raises the possibility of reversing the cycle. Our data indicate that this point of the cycle is an actual crossroad, considering the links of α-KG with HIF-1α stability [62], the possible derivation to lipid biosynthesis from cycle reversion, and its role in collagen biosynthesis and in the redox state through NADP to NADPH conversion. The levels of other selected metabolites measured by metabolomics confirm the significance of the metabolic reconstruction made from transcriptomic and ribosome profiling data (Figs. 5 and 6, Supplementary Figure S13 and Supplementary Table T6). Furthermore, it was described that IDH2 could acquire a neomorphic activity leading to the synthesis of D2-HG, an inhibitor of the PHDs involved in HIF-1α degradation [63]. The significant increase of fumarate (a competitive inhibitor of PDHs) and α-KG levels, together with the IDH2 switch, could be associated to triggering, maintaining, or enhancing the pseudohypoxia state.

Within quantitative and/or isozyme changes related to HIF-1α induction [60, 63–65], MKL1 ΔN200 cells showed enhanced expression of ENO1, PKM2, ALDOA, ALDOB, ALDOC, LDHA, and PDK1 in glycolysis, while no changes were detected in other HIF-1α targets. It is interesting to highlight that there are three switches in isozyme expression, which are not directly related to HIF-1α: (a) in glucose transporters; (b) in glycolysis, PFKM and PFKL to PFKP; and (c) in PDK isoform expression. These changes strongly suggest the involvement of other global metabolic regulatory factors that complement, modulate, or compensate for the action of HIF-1α.

We also wish to emphasize the notable increase in the expression of SLC2A4 glucose transporter. In most reported studies on cancer or metastatic cells, as well as in cells undergoing EM-like transition processes, an increase in glucose uptake has frequently been associated with changes in the expression of different transporters [59, 66–69]. SLC2A4 activation is dependent on insulin secretion and occurs mainly through activation of the PI3K/AKT pathway [70]. Its inhibition has recently been shown to block glucose uptake and inhibit AKT, critically affecting the viability of breast cancer cells [71].

The PFK-1 isoform switch appears to be a significant event since the PFK-1 step is a crucial control of glycolysis flux. The PFKP isoform, which was induced in MKL1 ΔN200 cells, frequently prevails over PFKM or PFKL in human cancer cells and in the EMT [72, 73]. PFKP has a lower affinity for the substrate; it is less sensitive to feedback inhibition by ATP and more sensitive to activation by fructose 2,6-bisphosphate (F2,6BP), which is the most potent allosteric activator of PFK-1 [74]. TIGAR (the expression of which is increased in MKL1 ΔN200 cells) lowers F2,6BP, resulting in the inhibition of PFK-1 and glycolytic activity. Furthermore, in response to hypoxia, posttranslational modification of PFK-1 results in inhibition of the kinase activity and redirected glycolysis flux towards PPP [72]. Then, the PFK-1 isoform switch appears as a critical step in glycolysis that diverts the metabolic flux towards the pentose phosphate pathway. It should be noted that TIGAR also contributes to 2-PG formation, which is associated with the parallel conversion of 1,3-BPG in 2,3-BPG. This finding is in agreement with the suggested role of TIGAR as a glycolytic shunt [75].

The other remarkable change corresponds to PDK isoforms, which are the key enzymes involved in the regulation of TCA feeding by pyruvate, through inhibition by phosphorylation of the catalytic subunit of the PDH complex. Great body of literature has been focused on the PDK family in recent years due to its role in crucial metabolic decisions and relation to cell proliferation, metabolic pathologies, and cancer [76, 77]. PDK1 and PDK3 play key roles in hypoxia adaptation, with both genes being induced by HIF-1α and cMyc [65, 73, 78–80, 87–89]. The enhanced expression of PDK4 in MKL1 ΔN200 cells constitutes an interesting finding. PDK4 has been described as a critical mediator of EMT [81] and associated with antiestrogen resistance in human breast cancer [82]. PDK4 transcription is regulated by numerous global metabolic regulators [83, 84]. In particular, ERRγ activates PDK4 transcription [85]. In MKL1 ΔN200 cells, ERRγ (ESRRG gene) expression is increased approximately 10-fold. Furthermore, untransformed human mammary MCF10 cells exhibit upregulated PDK4 upon matrix detachment, despite the action of ERRγ [76, 86].

The above considerations strongly suggest that multiple regulatory events should be considered to understand the metabolic transitions that occur in MKL1 ΔN200 cells: (a) enhancement of a pseudohypoxia context in which HIF-1α plays a pivotal role; (b) an effect associated with actual or pseudo matrix detachment, in which PDK4 would play a critical role; (c) the participation of different master regulatory factors that could function in a synergistic or balanced manner (particularly, the observed expression changes indicate the involvement of cMyc and p53 [87–90]. Taken together, the results show that changes in energy metabolism in MKL1 ΔN200 cells are compatible with what was described for cells undergoing an EMT. The metabolic rewiring includes enzymatic switches and pathway shunts, intervening on a previous well-established Warburg-like metabolic strategy, privileging glycolysis over OXPHOS. The impairment of the OXPHOS pathway and the redirection to PPP without increasing ribonucleotide synthesis, together with the allowed metabolic shunts associated with the altered TCA, are consistent with the strategies described for EMTs to escape anoikis, the apoptotic process triggered by matrix detachment [86, 91].

How can the adaptive process induced in MKL1 ΔN200 cells be explained? Decreases in cell numbers as well as the downregulation of genes involved in cell division, cell cycle phase transition and chromatin remodeling point towards a non-proliferative state. On the other hand, cell culture analysis showed an increase in cell size and in the glucose consumption/lactate production rate. This suggests that the high increase in glucose consumption is not only being used for energy purposes. Is it possible that the high glucose consumption is used as a source of intermediates for the generation of building blocks that allows cell size increase and remodeling? Gene expression patterns suggest an increase in protein biosynthesis. These findings, together with the overexpression of genes involved in the reorganization of actin cytoskeleton and secretory pathways, raise the question as to whether there is also a redirection of cell energy towards the formation of motile structures and the secretion of proteins besides cell size increase. Such is the case, for example, for quiescent fibroblasts that maintain high metabolic activity and direct it in part towards breakdown and synthesis of proteins and lipids, and in part towards secretion of extracellular matrix proteins [92]. The cellular microenvironment, which is mainly composed of extracellular matrix, can be essential in the determination of cell fate [93–95]. Differences in EMC composition can facilitate tumor progression and cell migration [96, 97]. Tumor cells contribute to defining and modifying the ECM composition during cancer progression [98, 99]. The ECM composition is sensed by several membrane receptors, including integrins and focal adhesion structures [100], in which MKL1 ΔN200 cells are enriched.

## Conclusion

This study reveals multiple regulatory events associated with a metabolic and translational machinery adaptation to achieve a new homeostasis state and favor cell survival. The expression profile is consistent with an epithelial to mesenchymal transition. During the transition, the synthesis of ribosomal proteins and that of many translational factors are upregulated and this appears to be regulated at the translational level. Moreover, the results indicate an increase in ribosome biogenesis and translation activity during the cellular EM-like transition. We also detected an extensive metabolic rewiring occurring in an already “Warburg-like” context, in which enzyme isoform switches and metabolic shunts merge to HIF-1α orchestrate regulation, thus suggesting its crucial role along with other master regulatory factors. Furthermore, the flux towards the pentose phosphate pathway is fully active without increasing ribonucleotide synthesis. Major concerted isoform switches involve glucose transporters, phosphoglucokinase, pyruvate dehydrogenase kinase, and mitochondrial isocitrate dehydrogenase. During this transition, cells arrest proliferation, increase in size, and strongly upregulate cytoskeletal and extracellular matrix proteins. The present work shows that ribosome profiling with RNA-Seq complemented with biochemical measurements is a powerful approach to unveil in-depth global adaptive cellular responses and the interconnection of regulatory circuits, which will be helpful for the identification of new therapeutic targets.

## Supplementary information

**Additional file 1:.** Supplementary Figure S1. Read alignments. Boxplots showing the percentages of aligned reads to different features from the ribosome profiling (RPF) and RNA-Seq (total RNA).

**Additional file 2:.** Supplementary Figure S2. Correlation of the mRNA read counts. Only genes over the detection limit of 1 rpkm are included. r, Pearson correlation coefficient.

**Additional file 3:.** Supplementary Figure S3. Correlation of the RPF read counts. Only genes over the detection limit 1 rpkm are included. r, Pearson correlation coefficient.

**Additional file 4:.** Supplementary Figure S4. Western blots. Western blots were performed as previously described (9,10) using the primary antibodies against MKL1 (ab14984) from Abcam, ERα (sc-543) and p-ERK (sc-7383) from Santa Cruz Biotechnology, ERK 1/2 (4695) from Cell signaling technology and p-mTOR (5536) from Cell Signaling Technology.

**Additional file 5:.** Supplementary Figure S5. Invasion assay. Cells were subjected to 3D spheroid invasion assay on Matrigel. The cells were seeded (5000/well) in 96-well plate with round bottom previously coated with matrigel and incubated for 4 days to allow spheroid formation. Taken up in Matrigel solution, spheroids were then seeded on the top of a matrigel cushion already formed in 96-well plates. Images were taken by microscopy (DMIRB-Leica). Scale bar: 50 μm.

**Additional file 6:.** Supplementary Figure S6. Cell growth, glucose consumption, lactate production and cell size. a) Viable cell number (filled symbols) and viability (empty symbols) for MCF7 control cells (green), MKL1 ΔN200 (blue), and MKL1 ΔC301 (red) cells. b) Glucose (filled symbols) and lactate (empty symbols) concentration. c) Cell size expressed in arbitrary units determined as light refracted in the FSC channel determined by flow cytometry. Cells were induced with tetracycline at time 0. Error bars represent standard deviation from experimental triplicate measurements.

**Additional file 7:.** Supplementary Figure S7. GO term enrichment analysis. Biplot showing the log2-fold TMM differences of RPFs (y-axis) and mRNA (x-axis) between MCF7 MKL1 ΔN200 and MCF7 control cells. Genes with expression changes driven by transcription regulation are shown in blue, genes with increased translation efficiency in red and genes with decreased translation efficiency in green. Color shades represent log10 p-values resulting from the differential translation efficiency analysis: light shades indicate high values while strong shades indicate low values. Genes were considered differentially expressed if p-value <0.01 and abs(FC) >2. The fold change cutoff value is indicated as a line. Summary of the GO term enrichment analysis performed with the different group of genes between MCF7 MKL1 ΔN200 and MCF7 control is shown. Selected GO classes with an overrepresentation are indicated. For genes with expression changes driven by transcription regulation upregulated and downregulated genes were used independently in the GO analysis. TMM: trimmed mean of M values.

**Additional file 8: **Supplementary Figure S8. GO term enrichment analysis. Biplot showing the log2-fold TMM differences of RPFs (y-axis) and mRNA (x-axis) between MCF7 MKL1 ΔC301 and MCF7 control cells. Genes with expression changes driven by transcription regulation are shown in blue, genes with increased translation efficiency in red and genes with decreased translation efficiency in green. Color shades represent log10 p-values resulting from the differential translation efficiency analysis: light shades indicate high values while strong shades indicate low values. Genes were considered differentially expressed if *p*-value <0.01 and abs(FC) >2. The fold change cutoff value is indicated as a line. Summary of the GO term enrichment analysis performed with the different group of genes between MCF7 MKL1 ΔC301 and MCF7 control is shown. Selected GO classes with an overrepresentation are indicated. For genes with expression changes driven by transcription regulation upregulated and downregulated genes were used independently in the GO analysis. TMM: trimmed mean of M values.

**Additional file 9:.** Supplementary Figure S9. microRNA signature analysis of genes with differential translation efficiency. miREM software results using genes with decoupled regulation between transcription and translation (groups (ii) and (iii) of genes).

**Additional file 10:.** Supplementary Figure S10. microRNA signature analysis of genes with decreased translation efficiency. miREM software results using genes with decreased translation efficiency (group (iii)).

**Additional file 11:.** Supplementary Figure S11. Translation efficiency of 5'TOP containing genes is significantly increased in MCF7 MKL1 ΔN200 cells. Gene set enrichment analysis (GSEA) showing the association of the differential translation efficiency to 5'TOP genes. The bar-code plot indicates the position of the genes on the efficiency data rank-sorted, with red and blue colors indicating over- and undertranslation efficiency in MCF7 MKL1 ΔN200 compared to MCF7 control cells, respectively. The enrichment plot for 5'TOP genes shows skewing to the left, indicating an increase of the 5'TOP translation efficiency in the MCF7 MKL1 ΔN200 cells. Significance statistics for GSEA is shown on top of the gene set enrichment plot.

**Additional file 12:.** Supplementary Figure S12. qPCR of selected genes. For qRT-PCRs, 3 replicates per sample were used. Polysomal fractions were not pooled but instead fractions 7-14 for all samples were used individually for gene expression analysis (24 points per sample). Expression was normalized to the pGEMEX-1 RNA spike-in control. Values were calculated from the quantitation cycle (Cq) using the formula relative quantity = 2 Cq(min)-Cq(sample). Boxplots of corrected Cq values, acquired by subtracting the difference of the Cq of the corresponding RNA spike Cq are shown.

**Additional file 13:.** Supplementary Figure S13. Schematic of glycolysis, TCA and PPP showing expression changes at the translational level of enzymes involved in the different steps. Changes in MCF7 MKL1 ΔN200 (a) and MKL1 ΔC301 (b) compared to MCF7 control cells. Fold-change (FC) values were taken from Supplementary File S3; only FC values higher than 0.5 (FDR < 0.01) were considered as significant. Blue: glycolysis; orange: PPP; pink: TCA. Arrow colors indicate the following: increase (blue: FC>1.0, light blue: 1.0>FC>0.5); decrease (red: FC>1.0, light red: 1.0>FC>0.5); grey: no significant changes; orange: variable changes within an enzyme complex. Values shown inside the small boxes indicate the fold change in the amount of each metabolite between both cell lines (Supplementary Table T6). Stars depict changes in the prevailing isoform. Steps associated with NADP reduction are indicated.

**Additional file 14:.** Supplementary Figure S14. Immunofluorescence detection of HIF1-a In situ immunofluorescence staining of HIF1-a in MCF7-control, MCF7-ΔN200 and MCF7 MKL1 ΔC301 cells. ER expression analysis is presented as a control.

**Additional file 15:.** Supplementary Table T1. Quality and alignment statistics. Each row represents one biological replicate. Under the quality statistics section, columns list the numbers and percentages of reads that do not pass the quality filters (i.e., reads that are too short, adaptor only reads or reads without adaptor) and the filtered ones used for subsequent steps of the analysis. Under the alignment statistics section, columns list the numbers and percentages of reads that align to the different kinds of features to which the read alignment was performed.

**Additional file 16:.** Supplementary Table T2. Summary of the expressed genes. Each row represents one biological sample. Columns list the number of genes expressed at the transcription level (in total RNA samples), at the translation level (in RPF samples) and at both levels. A gene is considered present in a sample if rpkm >1. A gene is considered expressed in one condition if rpkm >1 in the 3 replicates simultaneously.

**Additional file 17:.** Supplementary Table T3. Comparison of expression fold changes between RNAseq and microarray data for selected genes. Microarray data was obtained from Jehanno and Fernandez-Calero et al. (29).

**Additional file 18:.** Supplementary Table T4. Translation efficiencies. Polysomal fractionation was done as described (15) with some modifications. Polysomal fractions were not pooled but instead fractions 7-14 for all samples were used individually for study gene expression analysis by qRT-PCRs (3 replicates per fraction, 24 points per sample). Expression was normalized to the pGEMEX-1 RNA spike-in control. Values were calculated from the quantitation cycle (Cq) using the formula relative quantity = 2 Cq(min)-Cq(sample). The inverse of corrected Cq values (corrected Cq^-1^) for specific monosomes and polysomes fractions are shown on the left. Translation efficiencies with different criterias were determined as the fold change of the polysomes fractions vs the monosomes fractions.

**Additional file 19: **Supplementary Table T5. Specific rates of glucose consumption and lactate production. Data are mean ± SD from three biological replicates. *, *p* < 0,05.

**Additional file 20: **Supplementary Table T6. Metabolomic measurements of various metabolites. Metabolites were analyzed by liquid chromatography (LC)- mass spectrometry (MS) (LC-MS/MS) as described [29, 30]. Only significant average fold change values (*p* < 0,05) from seven technical replicates of three biological replicates are shown. NS: not significant. (2) Only two biological replicates were measured for Glucose 6-P and Oxaloacetate. (1) Only one biological replicate was measured for DHAP and ɑ-Ketoglutarate.

**Additional file 21:.** Supplementary File S1. Lists of genes with particular expression. Genes with high transcription and low translation comprise the group of red genes; genes with high transcription and high translation are designated blue; genes with high transcription and high but saturated translation are shown in green. In each list, genes that fulfill the condition in a particular sample are identified in gray. ND: not detected in the particular sample.

**Additional file 22:.** Supplementary File S2. Differential mRNA expression analysis.

**Additional file 23:.** Supplementary File S3. Differential RPF expression analysis.

**Additional file 24:.** Supplementary File S4. Differential translation efficiency analysis.

**Additional file 25:.** Supplementary File S5. GO term enrichment analysis between MKL1 ΔN200 and MKL1 ΔC301. The GO analysis was performed with differentially transcribed, differentially translated and differentially transcribed and translated genes separately. Selected GO terms used in the figures are highlighted in gray.

**Additional file 26:.** Supplementary File S6. GO term enrichment analysis between MKL1 ΔC301 and MCF7 control cells. The GO analysis was performed with differentially transcribed, differentially translated and differentially transcribed and translated genes separately. Selected GO terms used in the figures are highlighted in gray.

**Additional file 27:.** Supplementary File S7. GO term enrichment analysis between MKL1 ΔN200 and MCF7 control cells. The GO analysis was performed with differentially transcribed, differentially translated and differentially transcribed and translated genes separately. Selected GO terms used in the figures are highlighted in gray.

**Additional file 28:.** Supplementary File S8. Results of both total RNA and RPF of the differential expression analysis for the genes encoding components of the translation machinery. Genes that are significantly regulated are shown in red.

## Data Availability

The data sets supporting the results of this article are included within the article and its additional files. Raw data is available in the SRA database (https://www.ncbi.nlm.nih.gov/sra/) under accession number PRJNA499096.

## Abbreviations

1,3BPG: 1,3-Bisphosphoglycerate
2,3BPG: 2,3-Bisphosphoglycerate
2PG: 2-Phosphoglycerate
3PG: 3-Phosphoglycerate
5′TOP: RNA 5′ terminal oligopyrimidine motif
6PGL: 6-Phosphoglucolactone
6PG: 6-Phosphogluconate
ACO: Aconitase
ACLY: ATP-citrate lyase
Acta2: Actin (alpha 2)
ACTN: Actinin
a-KG: α-ketoglutarate
ALDO: Aldolase
ALDOA, ALDOB, ALDOC: ALDO isoforms
CCN: Cyclins
CD: Cluster of differentiation
CDS: Coding sequences
CML cells: Chronic myelogenous leukemia cells
CS: Citrate synthase
CSRP2: Cysteine and glycine-rich protein
CTGF: Connective tissue growth factor
D2-HG: D-2-hydroxyglutarate
DHAP: Dihydroxyacetone phosphate
DLAT: Dihydrolipoamide acetyltransferase (PDH component E2)
DLD: Dihydrolipoamide dehydrogenase (PDH component E3)
E2: 17-β-estradiol
E4P: Erithrose-4-phosphate
ECM: Extracellular matrix
EGLN: HIF prolylhydroxylase (PDH)
EIF: Eukaryotic initiation factor
EMT: Epithelial to mesenchymal transition
ENO: Enolase
EPCAM: Epithelial cell adhesion molecule
ER: Estrogen receptor alpha
ERBB2: Receptor tyrosine-protein kinase ERBB2
ERR: Estrogen-related receptor
F1,6BP: Fructose-1,6-bisphosphate
FBP1: Fructose-1,6-bisphosphatase 1
FBP2: Fructose-2,6-bisphosphatase 2
FCS: Fetal calf serum
FDR: False discovery rate
FH: Fumarate hydratase
FIH: Factor inhibiting HIF1
FLNA: Filamin A
FN1: Fibronectin 1
F6P: Fructose-6-phosphate
FSC: Forward scatter
FSP1: Fibroblast-specific protein 1
G3P: Glyceraldehide-3-phosphate
G6P: Glucose-6-phosphate
G6PD: Glucose-6-phosphate dehydrogenase
GAPDH: Glyceraldehide-3-phosphate dehydrogenase
GATA3: GATA binding protein 3
GLS: Glutaminase
GLUD: Glutamate dehydrogenase
GO: Gene ontology
GPD: Glycerol-3-phosphate dehydrogenase
GPI: Glucose-6-phosphate isomerase
HER2: Human epithelial growth factor receptor2
HIF1A-AS: HIF1A antisense RNA
HIF-1: hypoxia-inducible factor-1α
HK: Hexokinase
IDH: Isocitrate dehydrogenase
ITGA5: Integrin alpha-5 (fibronectin receptor subunit)
KRT8: Keratin-8
LARP1: La-related protein 1
LDH: Lactate dehydrogenase
MAPK: Mitogen-activated protein kinase
MCT: Monocarboxylate transporter
MDH: Malate dehydrogenase
MKL1: Myocardin-like protein 1
mTORC1: Mammalian target of rapamycin complex 1
NAD: Nicotinamide adenine dinucleotide
NADP: Nicotinamide adenine dinucleotide phosphate
NGS: Next generation sequencing
OGDH: Ketoglutarate dehydrogenase complex
OXPHOS: Oxidative phosphorylation
P53: Tumor protein p53
PCK: Phosphoenolpyruvate carboxykinase
PDH: Pyruvate dehydrogenase complex
PDH: Pyruvate dehydrogenase component E1
PDK: Pyruvate dehydrogenase kinase
PEP: Phosphoenolpyruvate
PFK: Phosphofructokinase
PFKM, PFKL, PFKP: PFK isoforms
PGD: Phosphogluconate dehydrogenase
PGLS: 6-Phosphogluconolactonase
PGK: Phosphoglycerate kinase
PGAM: Phosphoglycerate mutase
PHD: Prolyl hydroxylases
PHGDH: Phosphoglycerate dehydrogenase
PI3K/AKT: PI3K/AKT signaling pathway
PK: Pyruvate kinase
RPE: Ribulose-5-phosphate 3- epimerase
PPP: Pentose Phosphate Pathway
PR: Progesterone receptor
PRKN: Parkin
PRPP: Ribosylpyrophosphate
R5P: Ribose-5-phosphate
RL5P: Ribulose-5-phosphate
PRPS: Phosphoribosyl pyrophosphate synthetase
RP: Ribosomal proteins
RPFs: Ribosome protected fragments
RPIA: Ribose-5-phosphate isomerase A
RPS: Small subunit ribosomal protein
RPL: Large subunit ribosomal protein
S7P: Sedoheptulose-7-phosphate
SDH: Succinate dehydrogenase complex
SLC2A: Solute carrier family 2
snoRNAs: Small nucleolar RNAs
SRF: Serum response factor
SSC: Side scatter
SUCL: Succinate-coenzyme A ligase
TALDO: Transaldolase
TAGLN: Transgelin
TCA cycle: Tricarboxylic acid cycle
TIGAR: TP53-inducible glycolysis and apoptosis regulator
TJP: Tight junction proteins
TKT: Transketolase
TPI: Triose phosphate isomerase
TMM: Trimmed mean of M values
Ub: Ubiquitin
VHL: Von Hippel-Lindau factor
VTN: Vitronectin
Wnt5b: Wnt family member 5B
X5P: Xylulose-5-phosphate
