# Supplementary figures and images for: Fine-tuning the metabolic rewiring and adaptation of translational machinery during an epithelial-mesenchymal transition in breast cancer cells
Source: Cancer Metab. 2020 Jul 19;8:8. doi: 10.1186/s40170-020-00216-7 (PMC7368990; doi:10.1186/s40170-020-00216-7)

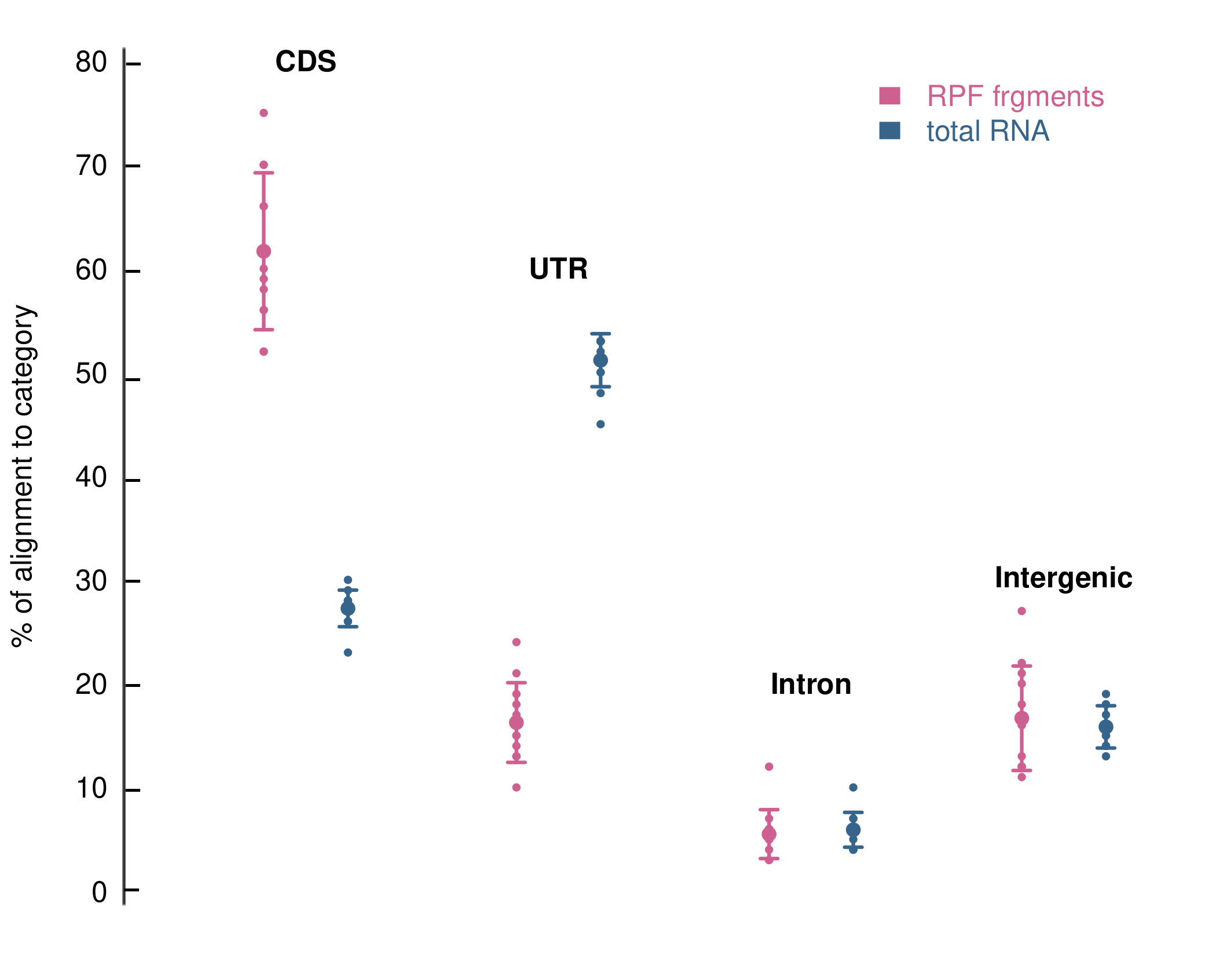

Supplement: Supplementary file 1 — Additional file 1:. Supplementary Figure S1. Read alignments. Boxplots showing the percentages of aligned reads to different features from the ribosome profiling (RPF) and RNA-Seq (total RNA). [file 40170_2020_216_MOESM1_ESM.tif]

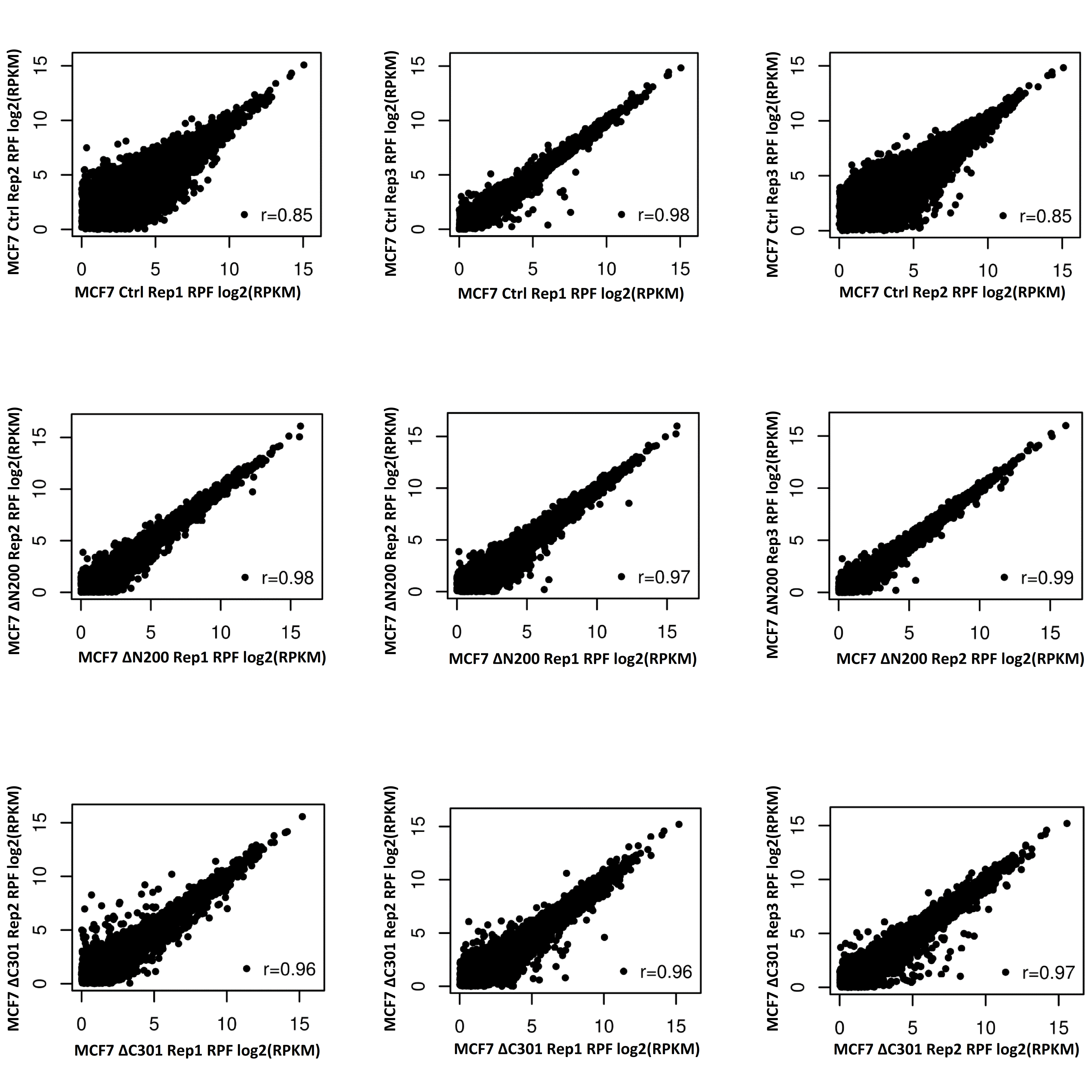

Supplement: Supplementary file 2 — Additional file 2:. Supplementary Figure S2. Correlation of the mRNA read counts. Only genes over the detection limit of 1 rpkm are included. r, Pearson correlation coefficient. [file 40170_2020_216_MOESM2_ESM.tif]

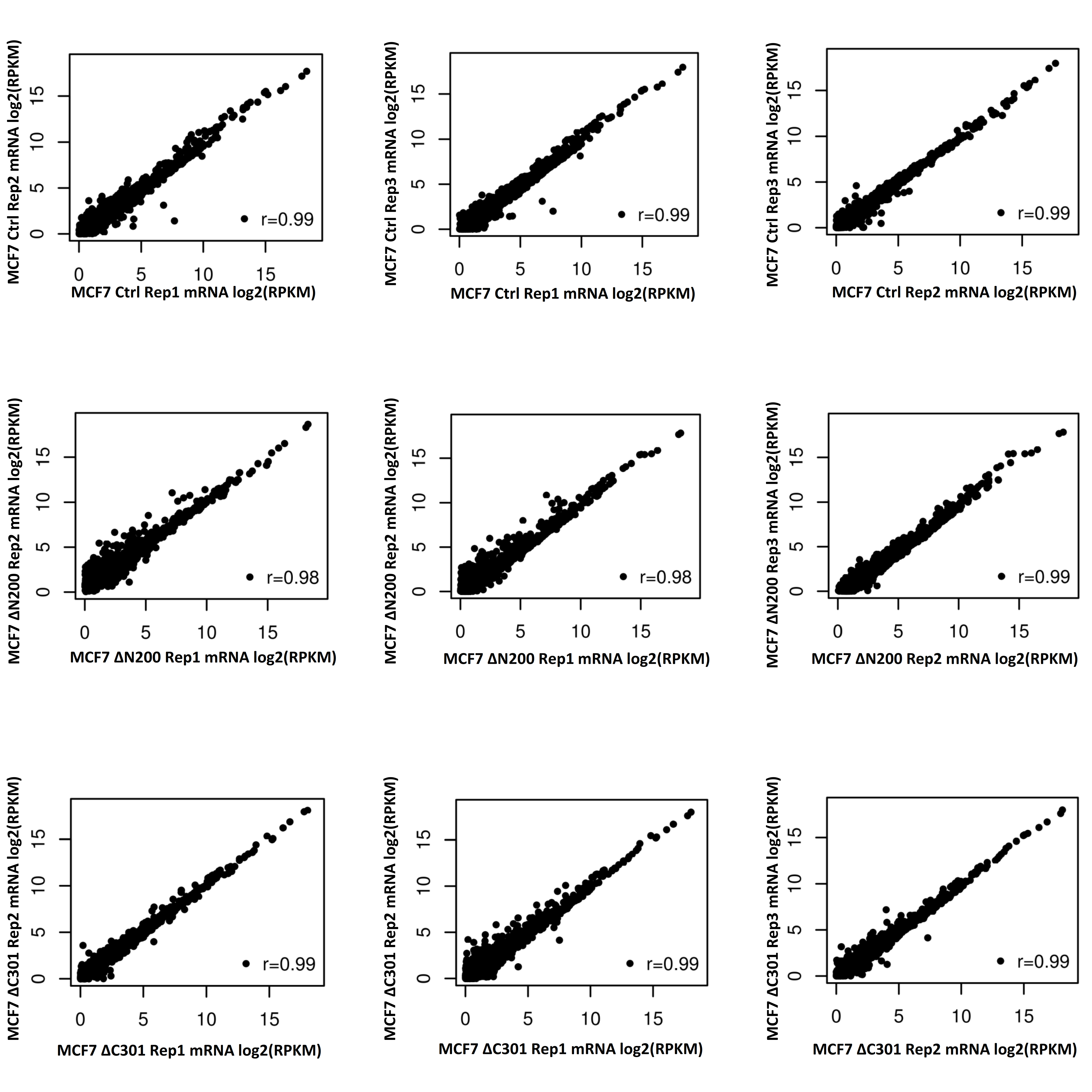

Supplement: Supplementary file 3 — Additional file 3:. Supplementary Figure S3. Correlation of the RPF read counts. Only genes over the detection limit 1 rpkm are included. r, Pearson correlation coefficient. [file 40170_2020_216_MOESM3_ESM.tif]

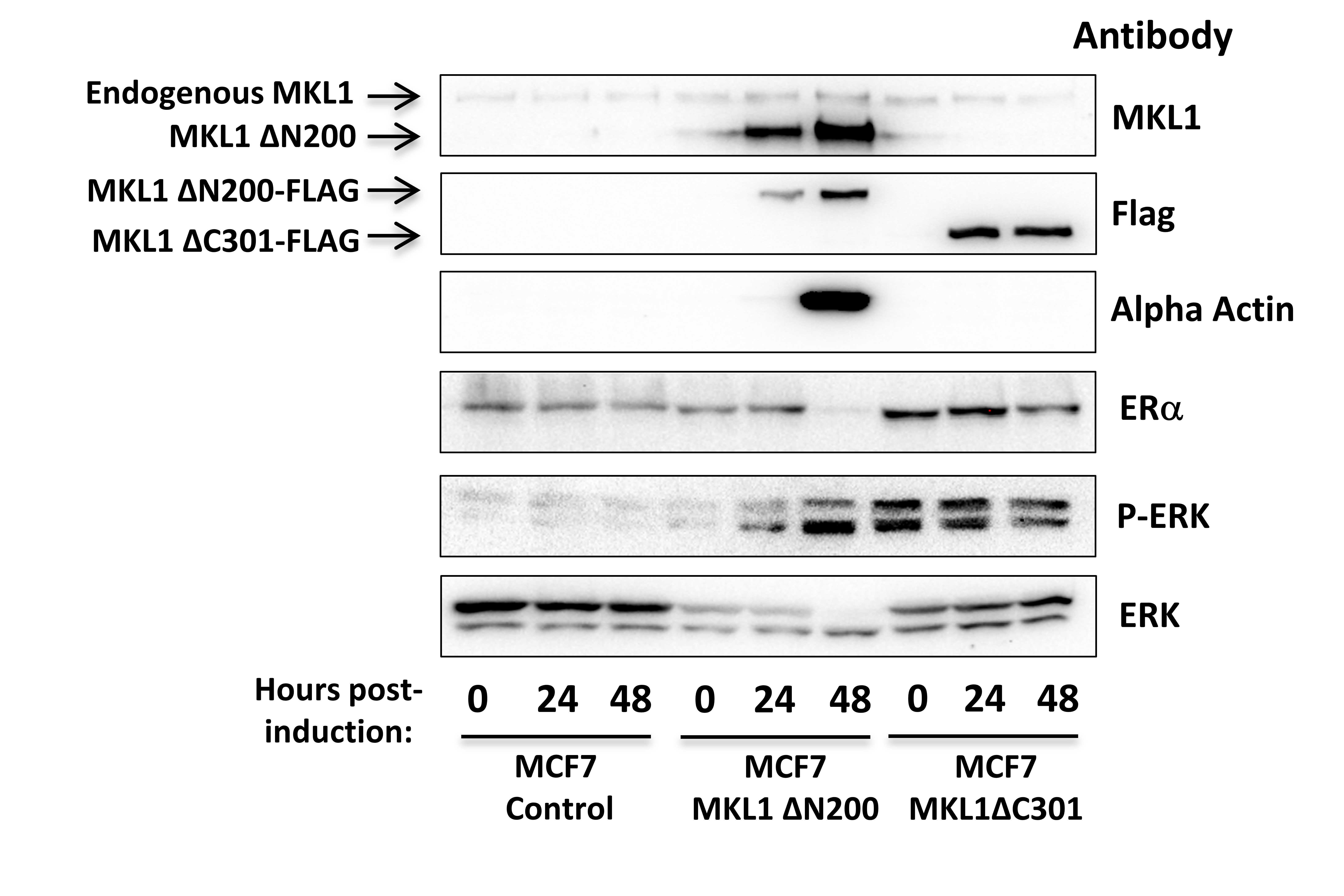

Supplement: Supplementary file 4 — Additional file 4:. Supplementary Figure S4. Western blots. Western blots were performed as previously described (9,10) using the primary antibodies against MKL1 (ab14984) from Abcam, ERα (sc-543) and p-ERK (sc-7383) from Santa Cruz Biotechnology, ERK 1/2 (4695) from Cell signaling technology and p-mTOR (5536) from Cell Signaling Technology. [file 40170_2020_216_MOESM4_ESM.tif]

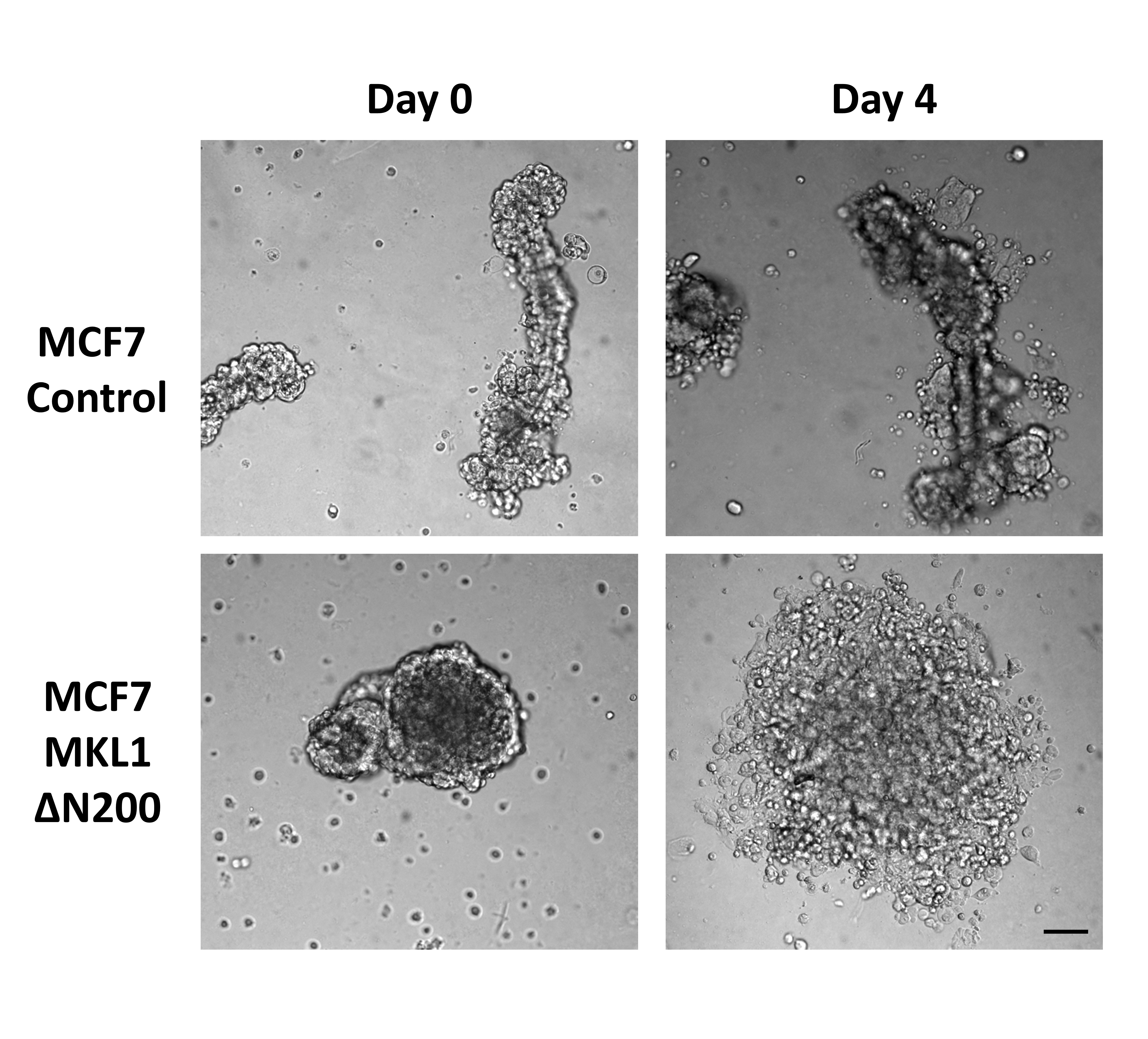

Supplement: Supplementary file 5 — Additional file 5:. Supplementary Figure S5. Invasion assay. Cells were subjected to 3D spheroid invasion assay on Matrigel. The cells were seeded (5000/well) in 96-well plate with round bottom previously coated with matrigel and incubated for 4 days to allow spheroid formation. Taken up in Matrigel solution, spheroids were then seeded on the top of a matrigel cushion already formed in 96-well plates. Images were taken by microscopy (DMIRB-Leica). Scale bar: 50 μm. [file 40170_2020_216_MOESM5_ESM.tif]

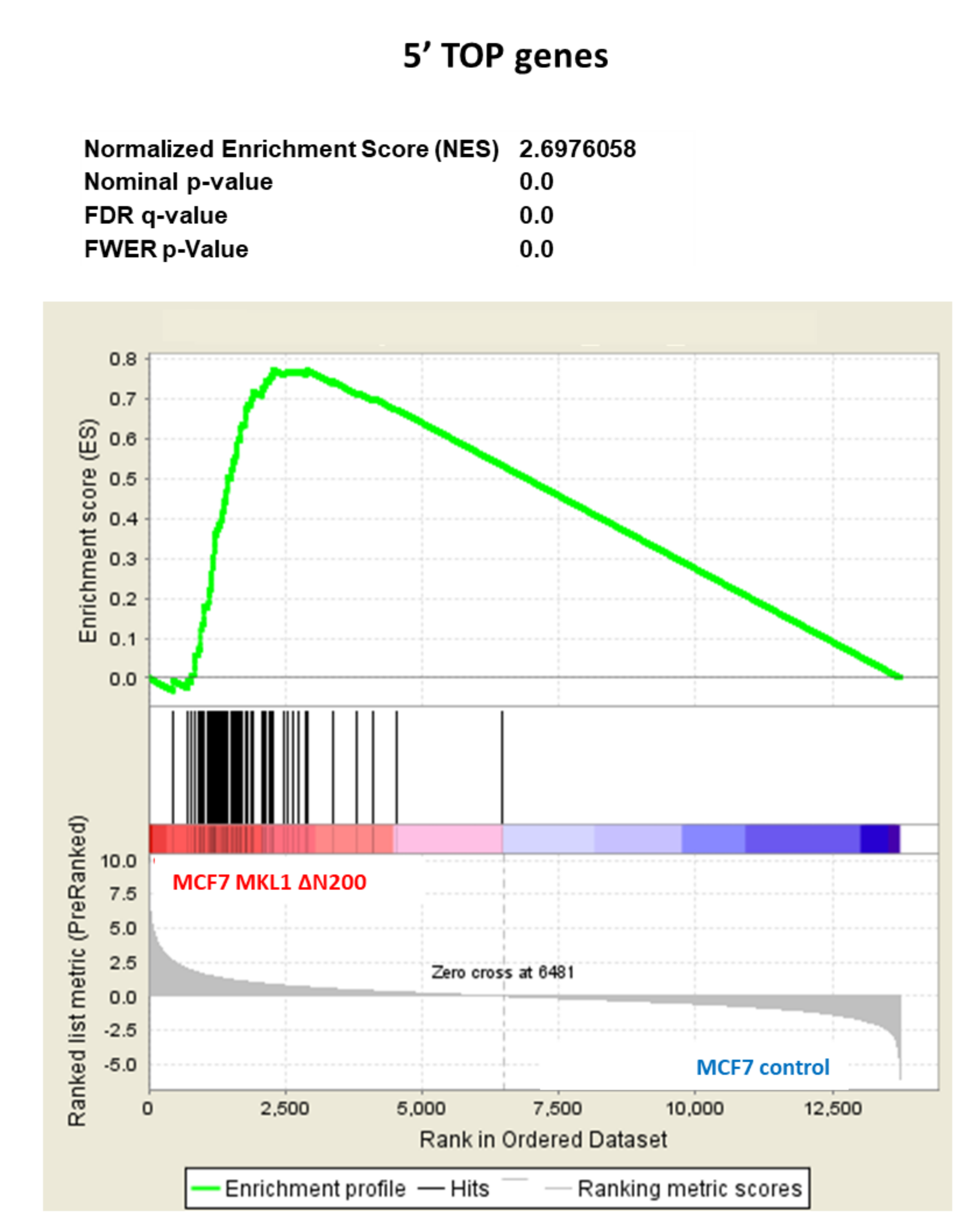

Supplement: Supplementary file 11 — Additional file 11:. Supplementary Figure S11. Translation efficiency of 5'TOP containing genes is significantly increased in MCF7 MKL1 ΔN200 cells. Gene set enrichment analysis (GSEA) showing the association of the differential translation efficiency to 5'TOP genes. The bar-code plot indicates the position of the genes on the efficiency data rank-sorted, with red and blue colors indicating over- and undertranslation efficiency in MCF7 MKL1 ΔN200 compared to MCF7 control cells, respectively. The enrichment plot for 5'TOP genes shows skewing to the left, indicating an increase of the 5'TOP translation efficiency in the MCF7 MKL1 ΔN200 cells. Significance statistics for GSEA is shown on top of the gene set enrichment plot. [file 40170_2020_216_MOESM11_ESM.png]

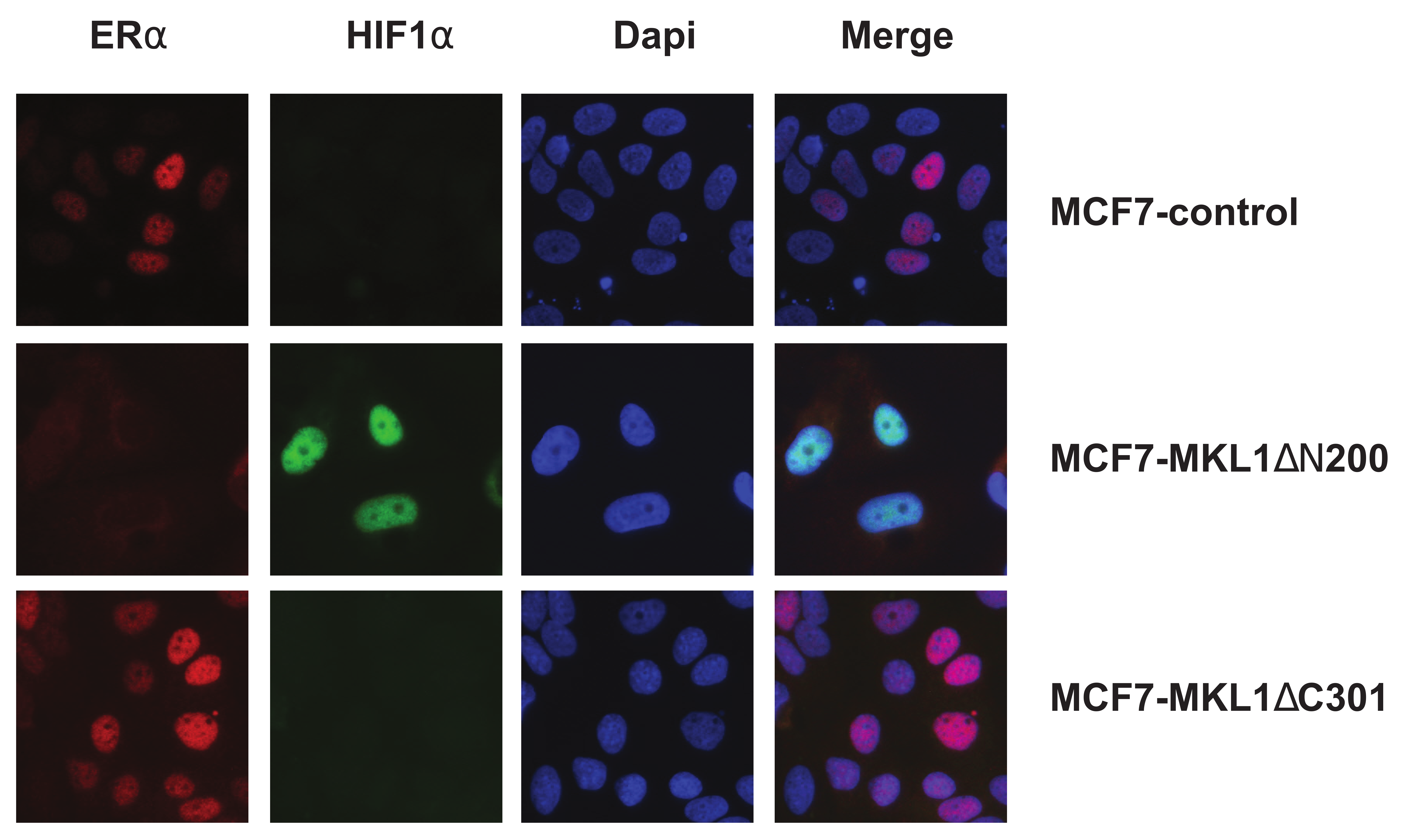

Supplement: Supplementary file 14 — Additional file 14:. Supplementary Figure S14. Immunofluorescence detection of HIF1-a In situ immunofluorescence staining of HIF1-a in MCF7-control, MCF7-ΔN200 and MCF7 MKL1 ΔC301 cells. ER expression analysis is presented as a control. [file 40170_2020_216_MOESM14_ESM.png]
